# Rapid one-step biotinylation of biological and non-biological surfaces

**DOI:** 10.1038/s41598-018-21186-3

**Published:** 2018-02-12

**Authors:** Stephen Henry, Eleanor Williams, Katie Barr, Elena Korchagina, Alexandr Tuzikov, Natalia Ilyushina, Sidahmed A. Abayzeed, Kevin F. Webb, Nicolai Bovin

**Affiliations:** 1 AUT Centre for Kode Technology Innovation, School of Engineering, Computer & Mathematical Sciences, Auckland University of Technology, Auckland, New Zealand; 2 Shemyakin & Ovchinnikov Institute of Bioorganic Chemistry, Russian Academy of Sciences, Moscow, Russian Federation; 3 FDA CDER, 10903 New Hampshire Avenue, Silver Spring, MD 20993 USA; 4 Optics & Photonics Research Group, Department of Electrical & Electronic Engineering, University of Nottingham, Nottingham, UK

## Abstract

We describe a rapid one-step method to biotinylate virtually any biological or non-biological surface. Contacting a solution of biotin-spacer-lipid constructs with a surface will form a coating within seconds on non-biological surfaces or within minutes on most biological membranes including membrane viruses. The resultant biotinylated surface can then be used to interact with avidinylated conjugates, beads, vesicles, surfaces or cells.

## Introduction

Biotinylation has for many years been used as important tool for attaching a variety of molecules to surfaces including cells, via the strong affinity association constant of 10^15^ M^−1^ with avidin. The attachment of biotin to non-biological surfaces generally requires multistep functionalisation of the surface while the attachment of biotin to cells and viruses usually involves covalently attaching biotin to random proteins at the cell surface^1^. As most cells and viruses are relatively fragile, exposure to chemical modification has the risk of affecting vitality, functionality and can modify antigenicity^2^.

Recently a series of water-dispersible amphipathic constructs known as function-spacer-lipids (FSLs) have been described for labelling cells, membrane viruses, and solid surfaces with a range of biological and non-biological markers under physiological conditions^3,4^. The basic structure of the FSL construct is typically a hydrophilic functional head; a biologically inert hydrophilic spacer which assists the construct to disperse in water and spaces the functional head away from the surface; and a lipid tail which makes the construct amphipathic and drives the spontaneous self-assembly on surfaces and insertion into lipid-based membranes^3^. Here we describe application of FSLs where the functional head is biotin. Terminology guidelines have been adopted (see Supplementary Note 3).

## Results

### Synthesis

**Figure 1:**
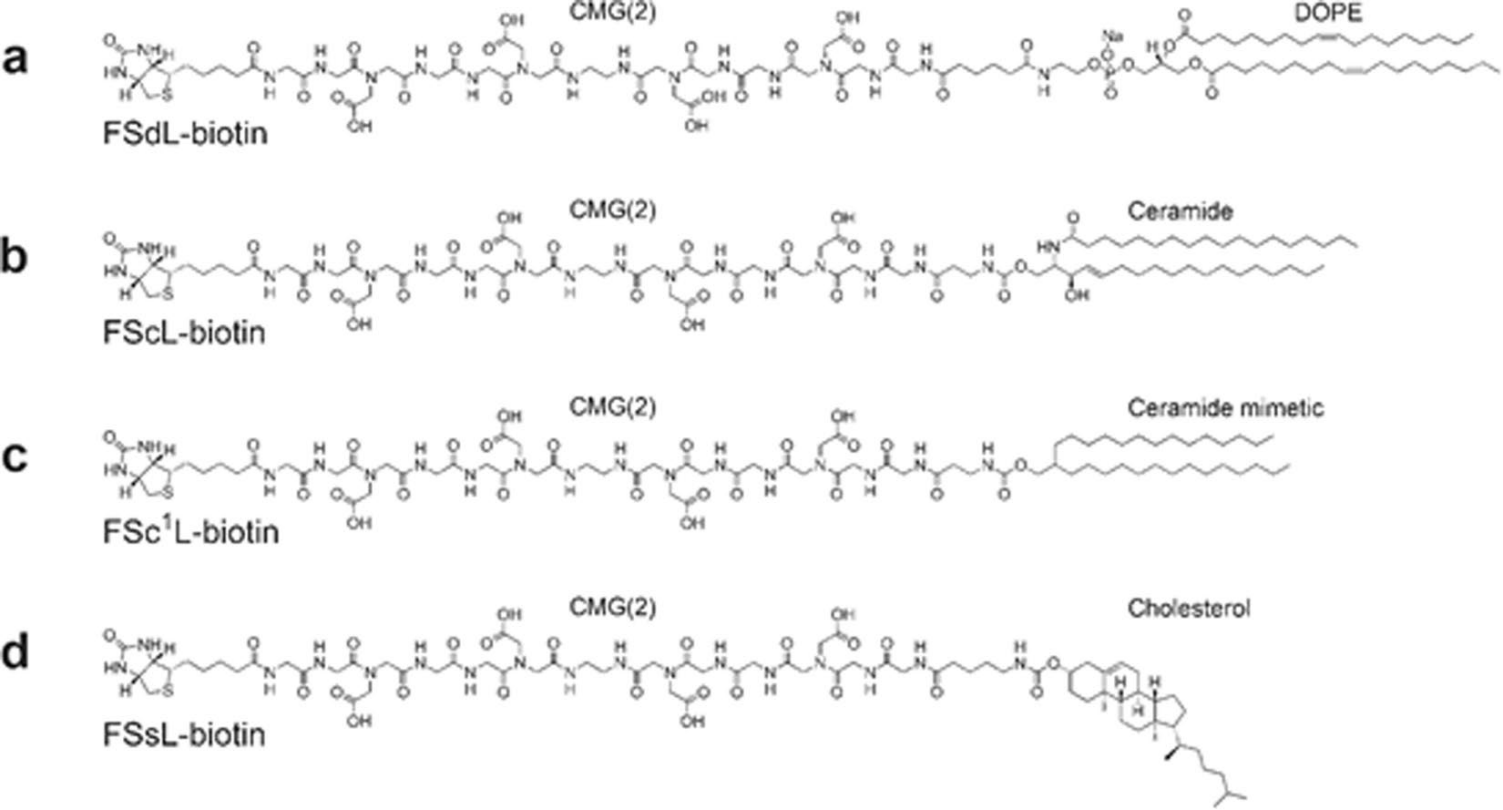
Chemical structures of FSL-biotin constructs with lipid variations. (**a**) FSdL: 1,2-O-dioleoyl-*sn*-glycero-3-phosphatidylethanolamine. (**b**) FScL: ceramide (synthesized chemically, identical to natural one). (**c**) FSc^1^L: ceramide mimetic, 2-(tetradecyl)hexadecanol. (**d**) FSsL: cholesterol. In order to describe the large variety and combinations of FSL constructs and the biological and non-biological surfaces and membranes they can modify.

FSL-biotin based on a negatively charged carboxymethylglycine (CMG(2)) spacer with a DOPE lipid tail (FSdL-biotin) (Fig. 1) is commercially available (Sigma-Aldrich, cat #F9182), but its performance characteristics are largely unreported^5–9^. For this evaluation we also synthesized several variants including two constructs with synthetic ceramide lipid tails (FScL-biotin), identical to the natural C18:0 ceramide, and one with a ceramide mimetic 2-(tetradecyl)hexadecanol (FSc^1^L-biotin). A construct with cholesterol (FSsL-biotin) was also synthesized according to published procedures and new synthesis developments^5^ (Supplementary Notes 1a–d). In addition, a variation with an uncharged O-methylated CMG(2) spacer was also prepared (Supplementary Note 1a). For the uses described in this paper all FSL-biotin constructs (Fig. 1a–d) performed well, except the uncharged spacer construct which had limited solubility in water and aqueous buffers.

### Biotinylation of cells

There are many methodological variations that can be tailored and used to prepare kodecytes (i.e. cells modified with Kode constructs), each with subtle differences in the rate and amount of FSL construct that will insert into the cell membrane. Provided the same procedure and variables are used for all cells of a specific type and quality, the experimental results will be comparable between kodecytes prepared on different occasions. Typically assay controls are prepared at the same time as kodecytes using the same cells except they are incubated either with the FSL diluent (without FSLs) and ideally also with a benign FSL (of similar structure but with no activity).

**Figure 2:**
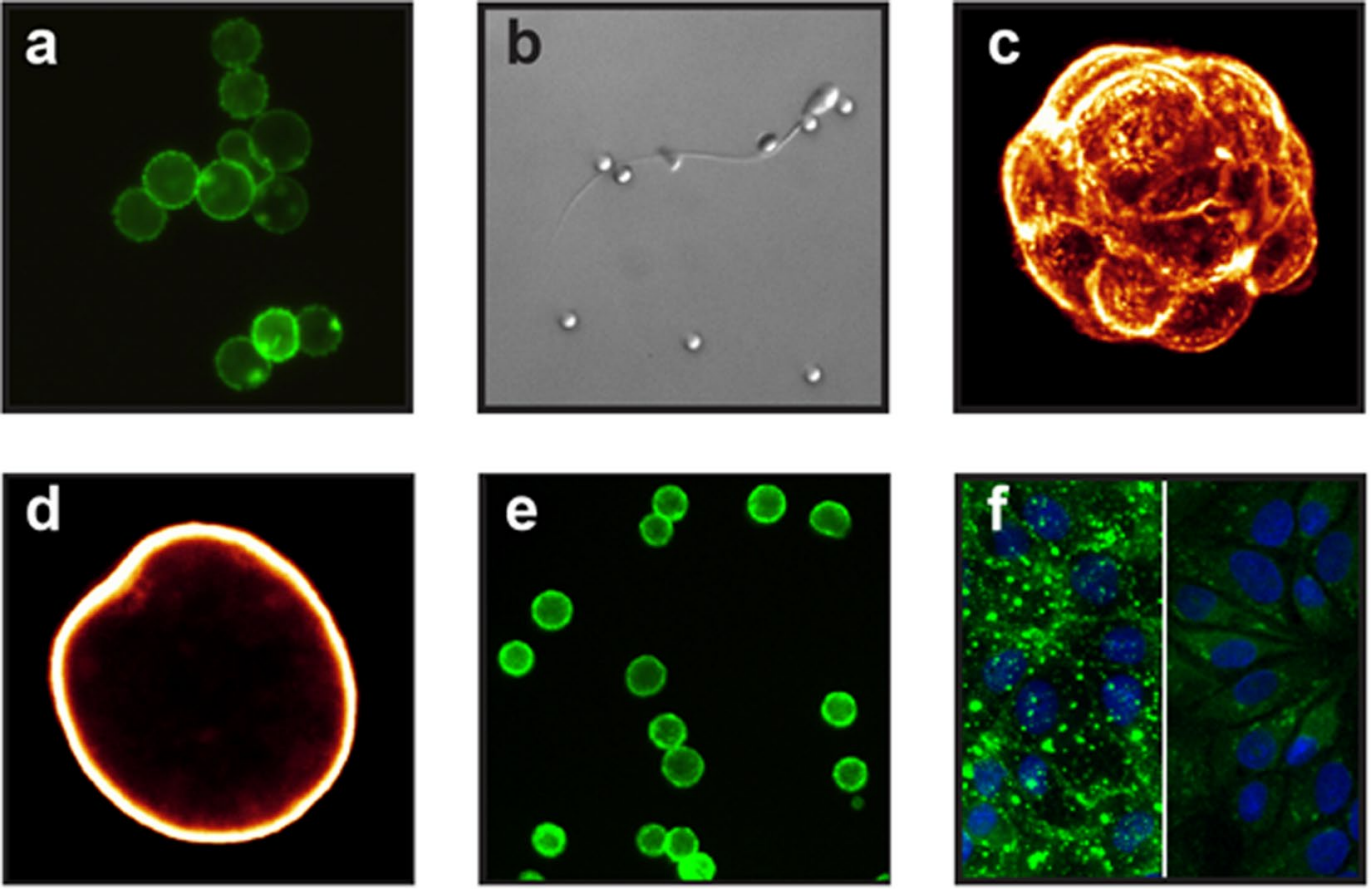
FSL-biotinylation of biologicals with FSLs, examples. Generic methods for (**a–f**) are as described in Methods. (**a**) Fluorescent image of human red cells incubated in FSc^1^L-biotin (5 µM) and visualised with NeutravidinDyLight488 (100 µg/mL), (400× magnification). (**b**) Light image of motile human spermatozoa incubated with FSdL-biotin (50 µM) then immobilized with Dynabeads M-280 Streptavidin (2.8 µm) (400× magnification). (**c**) Confocal image of murine embryo with zona pellucida removed, incubated with FSdL-biotin (50 µM) and visualized with Avidin Alexa Fluor488 (2 µg/mL) then fixed in 4% formaldehyde. (**d**) Confocal image of zona pellucida prepared as in (**c**). (**e**) Fluorescent image of epithelial RL95 cells incubated with FSdL-biotin (250 µM) and visualized with Avidin Alexa Fluor488 (100 µg/mL) (200× magnification). (**f**) Fluorescent image of MDCK cells infected (for 4 h at 37 °C) with A/California/04/09 H1N1 influenza virus labelled with 25 µM FSdL-biotin for 1 h. Left panel, infected cells; right panel, mock-infected cells. Cells were fixed with 4% paraformaldehyde and stained with Alexa Fluor488 Streptavidin. Viruses are stained in green; nuclei—in blue (DAPI). Viruses are detected both on cell surface and inside cells (400× magnification).

Biotinylation of intact cellular biological membranes with FSL-biotin to create *biotin-kodecytes* involved contacting a suspension of cells with a buffer containing FSL-biotin and incubating at 37 °C for at least 30 min. Concentration dependent modification occurred with dilutions of FSL-biotin over the range of 0.5–50 µM and the resultant biotinylation was easily detected with fluorescently labelled avidin (Fig. 2). Biotin-kodecytes were able to maintain their biotin labels for time-periods related to their rate of membrane activity (vitality) and the environment in which they were exposed, usually for at least 24 h or more^8,9^. Extensive washing did not effect on the level of labelling on biotin kodecytes. Non-viable and dead cells are strongly labelled and retain their label longer than viable cells, thus indirectly providing a simple method of determining viability and separating viable cells from non-viable cells.

All forms of avidin were able to effectively convert biotin-kodecytes into avidin-kodecytes however there were sometimes different performance characteristics depending on the innate reactivity of the avidin type with the surfaces used. Modification of membrane viruses with FSL constructs to create so-called kodevirions^10^ was achieved by contacting a suspension of virus particles with a buffer containing FSL-biotin and incubating at 37 °C for 30–60 min^7^. Biotin kodevirions (Fig. 2f) were functional and able to maintain their biotin labels for time-periods related to their environment. Moreover, no differences were observed in growth rates between biotin kodevirions and their respective unlabeled counterparts at any of the post-infection time points *in vitro*, indicating than non-destructive non-covalent modification of viral membranes with FSL constructs do not affect viral replication fitness^7^. General recommendations for FSL constructs handling see in Supplementary Note 4.

### Coating of surfaces

The ability of FSL-biotin to label non-biological surfaces was evaluated by applying FSL-biotin constructs to a large variety of non-corroding surfaces as previously done with glycan-FSLs^3^. It was found that all tested non-biological surfaces were modified with FSdL-biotin, including materials such as plastics, paper, rubbers, metals, and composites, whether in a planar, spherical or fibrous/weave format including nanofibres (Supplementary Note 2).

**Figure 3:**
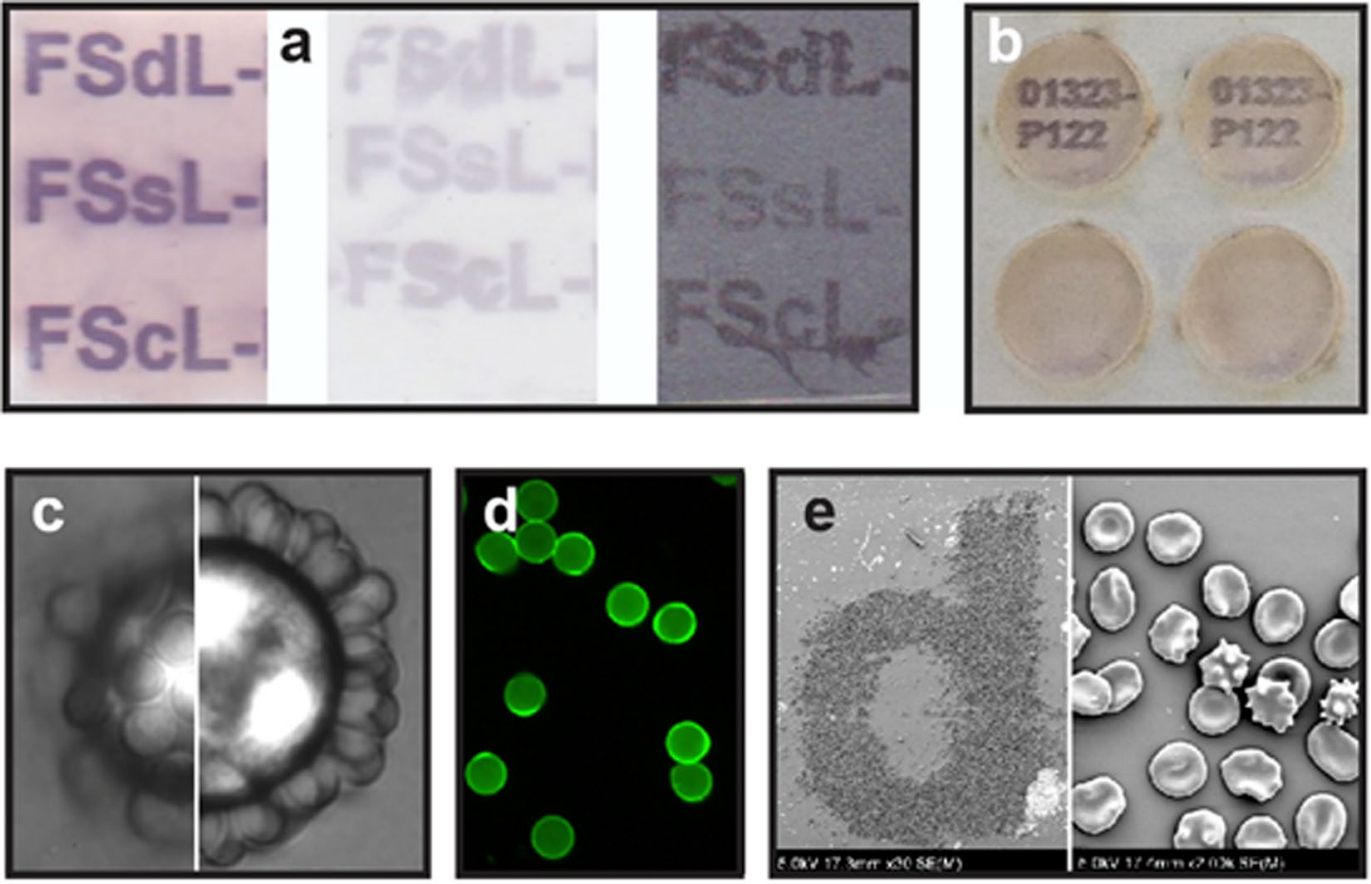
FSL-biotinylation of non-biological surfaces, examples. Generic methodologies are as described in Methods. (**a**) Inkjet printing of three different FSL-biotin constructs (Fig. 1) on three different materials; paper (left), polyester (middle) and 316 stainless steel (right), visualised by enzyme assay. (**b**) Immobilisation via streptavidin of a biotinylated HLA protein onto inkjet printed FSL biotin characters on paper, visualised by enzyme immunoassay, with negative reactions (lower wells) in the absence of biotinylated HLA protein. (**c**) Two focal planes of red cell biotin kodecytes captured onto biotin koded polycarbonate microspheres (20 µm) via streptavidin bridging, light microscopy (1000× magnification). (**d**) Biotin koded polycarbonate microspheres (20 µm) visualised by Avidin AlexaFluor®488 fluorescent microscopy (200× magnification). (**e**) FSdL-biotin printed on polyester visualised by capture of red cell biotin kodecytes *via* neutravidin bridging, left (30× magnification) and right (2000× magnification), SEM.

The primary surface detection method used was an enzyme assay with alkaline phosphatase labelled streptavidin (Fig. 3a). Alternatively, reactions could be visualised with biotinylated cells captured via avidin microspheres (Fig. 3c) or planar surfaces (Fig. 3e), or by an enzyme immunoassay (EIA), where a captured protein (*via* streptavidin) is visualised with specific antibodies and secondary reactions (Fig. 3b), or with fluorophore labelled avidin (Fig. 3d). Not all variations of avidin^11^ (e.g. avidin, neutravidin, streptavidin) are equally suitable for all surfaces and kodecyte combinations and optimization is required.

Factors influencing FSL surface coating and its stability were also evaluated primarily on stainless steel, polyester and paper surfaces (Fig. 3a), with results almost identical to those obtained with the glycan FSL constructs^5^. Composition of the FSL lipid tail, concentration (1–50 µM), contact time and method of application (inkjet printing, painting or immersion) were all found to subtly or significantly influence the quality and stability of the coating on different surfaces. The time required for an FSL-biotin construct to label a non-biological surface could be as short as 5 seconds, although generic methodology used 10–30 min. Modified surfaces did not require drying before use, but were usually air-dried, and were stable unprotected for at least 8 months at ambient temperature. FSL-biotin surfaces when contacted with water, saline or serum were stable for at least 12 hours, but the FSL-biotin construct could be removed with detergents with concentrations >1% or with solvents such as 70% methanol. In general, all surfaces were able to be modified by FSL-biotin adequately for most analyses, although some materials had better labelling and stability performance characteristics than others. The kinetics of the coating, as well as the formed layers stability for washing were evaluated using the SPR method (see Supplementary Note 5).

## Discussion

FSL-biotin is able to efficiently label almost all non-biological surfaces with a biotin coating that is sufficiently robust to immobilise proteins and cells (under gentle shear conditions). In addition, mammalian cells can be effectively labelled in a dose dependent manner with biotin, and the resultant biotin-kodecytes are able to interact (*via* avidin) with biotinylated proteins, non-biological surfaces and other cells. Similarly, membrane virions and liposomes were effectively labelled. There is no evidence of a loss in functionality or vitality of biotin-kodecytes^8,9^ or kodevirions^7^ and they can be used both for *in vitro* and *in vivo* studies. For general use the FSL-biotin based on the DOPE lipid tail (Fig. 1a) performed well, and unless a specific need exists there was no significant advantage of using other lipid variations (e.g. sterol or ceramide). Nevertheless, we expect that synthesised versions (Fig. 1b–d) will be required for design of membranes where presentation of the FSL (head/tail ratio) is important, and when the type of lipid tail affects the locating of the construct within a lipid membrane. FSL-biotin labelling is convenient and harmless method for temporary biotinylation of biological surfaces, and a rapid comprehensive method for temporary biotinylation of non-biological surfaces. It can be used either alone or with other existing methods. FSL-biotin together with other non-biotin FSLs, have the potential to expand research opportunities.

## Methods

### Biotin-kodecytes

The following method, together with cell-specific caveats, can be tailored to fit with any living or dead cell (including those modified with glutaraldehyde fixation before or after koding).

- Prepare an FSL-biotin stock solution in a lipid and detergent free buffer (e.g. PBS or serum-free cell culture media) at a concentration of 1 mM.
- It can be prepared in water, but may degrade upon 4 °C storage, and should be stored frozen as aliquots. Repeated freeze-thawing appears to have no effect on construct stability.
- Prepare the working FSL solution by diluting stock FSL-biotin solution in buffer (as above). The optimal dilution used will need to be experimentally determined for each particular use, but will usually be in the range of 1–100 μM (2–200 μg/mL), with typical concentrations for FSL-biotin intended for biological membranes being in the range of 10–50 μM. If necessary, other non-biotin FSL constructs can also be added to the same solution for simultaneous insertion.
- Next, prepare the cells intended for modification into kodecytes. Wash them free of serum lipids (e.g. at least 2 × in PBS) and make as a cell suspension of any known concentration in the same media as the FSL solution has been prepared. Typically, 50–80% cell suspensions are used as they can be delivered accurately by pipette; however a single cell can be modified if transferred to a solution of FSL constructs.
- Place one part of FSL solution into a test tube. Add up to one part of cell suspension to the one part of FSL solution. Mix and incubate, time and temperature will be that which is compatible with the cell being modified and can range from 4–40 °C and from 10 min up to several days. Typically, mammalian cells will be adequately modified after 30–120 min at 37 °C. The lower the temperature, the longer that may be required to achieve the same level of cell surface modification achieved at a higher temperature. Note: FSL constructs may become internalised by some cell types over time. Once the incubation is complete, transfer the newly formed kodecytes to an FSL free solution. There is no requirement to wash (or transfer) the kodecytes if low concentrations of FSL were used, but this is usually done to ensure no free FSLs are present in the solution phase, and to stop the reaction proceeding during storage if short incubation times are used. The solution receiving the kodecytes can be serum or contain lipids, however stability of the membrane bound FSLs may be reduced.

Avidin kodecytes are prepared from biotin-kodecytes by adding avidin in excess. The three common forms of avidin (egg avidin, streptavidin & neutravidin, collectively termed avidin) routinely available can all be used. The level of avidin on the kodecyte is directly controlled by the amount of FSL-biotin on the biotin-kodecyte and therefore needs not be directly described. Avidin-kodecytes will not self-aggregate provided all (most) biotin residues are saturated with avidin, this can be achieved by using avidin in excess.

- Prepare biotin-kodecytes (typically < 50 μM) and wash them once into the same buffer as is intended to prepare the avidin solution. Dilute the biotin-kodecytes to a <10% cell suspension. Prepare a solution of 7.5 μM avidin in PBS or other biocompatible (serum/detergent free) media. Add one part of avidin solution to one part of biotin-kodecytes, mix and incubate for 30 min at RT. Wash away unbound avidin (by centrifugation) with PBS.

### Koded surfaces

Almost any surface can be modified with FSL constructs and although the methods of application maybe different, all are essentially the same, and only involve contacting a solution of FSL with a surface. If the application of the FSL is to a non-biological surface, biologically incompatible solvent systems may be used (in ethanol, methanol and isopropanol:water mixes of less than 70%). If water (without salts) is used, the construct solution should be used within 24 h to reduce the risk of FSL degradation^3^.

Typical concentrations for FSL-biotin are in the range of 0.01–50 μM. If layering occurs, then upper layers may be more easily lost (e.g. by washing) to the environment than the primary layer in contact with the surface. It is for this reason that it is recommended the koded surfaces be washed prior to use (e.g. prior to reacting with avidin). Additionally, each different method of application, concentration of FSL, mixtures of FSLs, solvent surface tension and ability to wet the surface, air-solvent interfaces (when dipping), rate of drying (or not drying) and other factors may affect assembly of the FSL on a particular surface and can create edge layering artefacts. The subsequent stability/retention of an FSL surface coating may thus depend on all the above factors and the time, temperature and the environment the koded surface is exposed^6,7^.

Prepare an FSL-biotin solution at an appropriate concentration (typically 50 μM or less is suitable for most applications including inkjet printing). Most surfaces require no prior preparation unless they have an unbound coating on them, such as an oil film (which may be present on metal sheeting) or particulates. If required, wash the surface clean with an appropriate solvent. Drying of the surface prior to application is not necessary.

Application of the FSL to the surface can be by one of several of the following techniques:

#### Dipping/immersion

Prepare a vessel containing a solution of FSL and place the surface (planar or microspheres) to be modified into the solution. After a short incubation, or immediately remove the surface. A washing step (before or after drying) may be undertaken. Either allow the surface to dry or transfer to a new buffer for storage.

#### Brushing/spotting/spraying/drying

Use a paintbrush, dropper, pipette or spray unit to apply the FSL to a surface (including the wells of a microplate). Either allow the surface to dry or transfer to a new buffer for storage. A washing step may be undertaken if desired.

#### Inkjet printing

A piezoelectric inkjet printer (e.g. Epson Stylus T21) can be used to print FSLs^12^. Constructs are also compatible with thermal printers. Sponge-less, refillable cartridges are used, and can be volume capacity reduced by partially filling with an inert resin. Cartridges are cleaned with 70% ethanol and deionised water before filling with FSL. Solutions containing 50 μM of FSL-biotin were prepared for printing in PBS or water. Bromophenol blue dye can be included in the solution at a concentration of 0.5% to enable printed area to be observed—this dye being lost during subsequently washing procedures. The colour management of the printer was adjusted to print the desired solution from the correct cartridge, and prevent the mixing of inks (different FSL’s) in other cartridges. After inserting the FSL-loaded cartridge the printer was primed by printing solid blocks of colour, which removed air bubbles and primed the printing solution through to the print head. If the membrane to be printed was too fragile to allow passage through an inkjet printer (e.g. nano fibres, gold sheet), it could be stabilised on a support surface or captured onto a single side of a document laminating film (backing)^12^. After printing the koded surfaces could be used immediately or stored at RT for many months. After printing the inkjet print-head can be washed with water and 70% methanol:water, or preferably replaced or restricted to use with a single type of FSL.

### Avidin-koded surfaces

Similarly, to creating avidin-kodecytes a surface can be easily avidinylated by simply flooding the biotin koded surface with avidin. Essentially the surface is first prepared with an FSL-biotin coating (as above), washed 2–3 times with water or PBS to remove/reduce any layering of FSL’s then reacted with an avidin solution. If using microspheres, avidin should be in excess to reduce the possibility of aggregation due to crosslinking. Modified surfaces can be stored in PBS at 4 °C for at least several months.

### Capturing biotinylated proteins onto avidin-koded surfaces

Biotin + avidin koded surfaces (planar, fibrous or microspheres) can be used to capture biotinylated proteins (e.g. Fig. 3d). Avidin koded surfaces are first prepared as above. After (optional) blocking for 30 min with 2% BSA in PBS, biotinylated protein (including biotinylated IgG) appropriately diluted in 2% BSA, is flooded onto the surface and allowed to react with the FSL-biotin + avidin complex for 30 min at RT. The surface is then washed 6 times with PBS and is ready for visualisation with antibodies in an EIA assay or reacting with ligands or cells.

### Capturing kodecytes (or koded microspheres) onto biotin/avidin koded surfaces

To capture biotin-kodecytes (or biotin microspheres) onto avidin + biotin koded surfaces or alternatively to capture avidin + biotin-kodecytes (or microspheres) onto biotin koded surfaces (e.g. Figs 2b and 3b,c). Not all kodecytes or koded microspheres will interact with koded surfaces, and variations in interactions and binding may be observed depending on experimental conditions.

### Detection of FSL-biotin with labelled avidin (enzyme/fluorophore)

The detection of FSL-biotin on koded surfaces and/or kodecytes can be via the use of alkaline phosphatase labelled avidin (usually streptavidin) precipitation of the chromogenic substrate NBT/BCIP (e.g. Fig. 3a) or fluorophore labelled avidin (e.g. Fig. 3d).

#### Precipitating chromogenic substrate

Biotin koded surfaces are blocked with 3% BSA-PBS for 30 min then decanted. Onto each surface was flooded with streptavidin alkaline phosphatase (Sigma S2890) appropriately diluted in BSA-PBS and then incubated for 30 min at RT. BSA-PBS used as a negative reagent control. Following incubation plates were washed 6 times by immersion for 20 sec in beakers of fresh PBS. Precipitating chromogenic substrate (NBT/BCIP stock solution, 18.8 mg/mL nitro blue tetrazolium chloride and 9.4 mg/mL 5-bromo-4-chloro-3-indolyl phosphate toluidine salt in 67% DMSO (v/v); Roche, Mannheim, Germany) was diluted 1:50 in substrate buffer (100 mM Tris, 100 mM NaCl, 50 mM MgCl_2_, pH 9.5) and the surface was flooded with substrate. Surfaces were incubated at RT for 2–10 min and the reaction was stopped by rinsing with water. Developed plates were allowed to air dry and can be stored indefinitely unprotected at RT. The rare limitation of this technique is false negative results may occur if final precipitate is unable to be retained at the surface.

#### Fluorescence

Biotin koded surfaces (optionally blocked with 2% BSA) are flooded with streptavidin fluorophore (AlexaFluor 488) appropriately diluted (usually 0.1 mg/ml) in PBS and then incubated for 30 min at RT. Following incubation, the surfaces were washed 3 times with PBS before viewing under UV.

## Electronic supplementary material

Supplementary Note 1

Supplementary Note 2

Supplementary Note 3

Supplementary Note 4

Supplementary Note 5
