# Supplementary material for: Rapid one-step biotinylation of biological and non-biological surfaces
Source: Sci Rep. 2018 Feb 12;8:2845. doi: 10.1038/s41598-018-21186-3 (PMC5809608; doi:10.1038/s41598-018-21186-3)
Supplement: Supplementary file 1 — Supplementary Note 1 [file 41598_2018_21186_MOESM1_ESM.pdf]

Rapid one-step biotinylation of biological and non-biological surfaces

Stephen Henry[1*], Eleanor Williams[1], Katie Barr[1], Elena Korchagina[2], Alexandr Tuzikov[2], Natalia Ilyushina[3], Sidahmed A. Abayzeed[4], Kevin F. Webb[4], Nicolai Bovin[1,2*]

[1]AUT Centre for Kode Technology Innovation, School of Engineering, Computer & Mathematical Sciences, Auckland University of Technology, Auckland, New Zealand.

[2]Shemyakin & Ovchinnikov Institute of Bioorganic Chemistry, Russian Academy of Sciences, Moscow, Russian Federation

[3]FDA CDER, 10903 New Hampshire Avenue, Silver Spring, MD 20993, USA

[4]Optics & Photonics Research Group, School of Electrical & Electronic Engineering, University of Nottingham, United Kingdom

Supplementary Note 1. **Synthesis.**

Supplementary Note 1a. **Synthetic scheme and detailed protocol for synthesis of FSdL-biotin and corresponding neutral-spacer construct.**

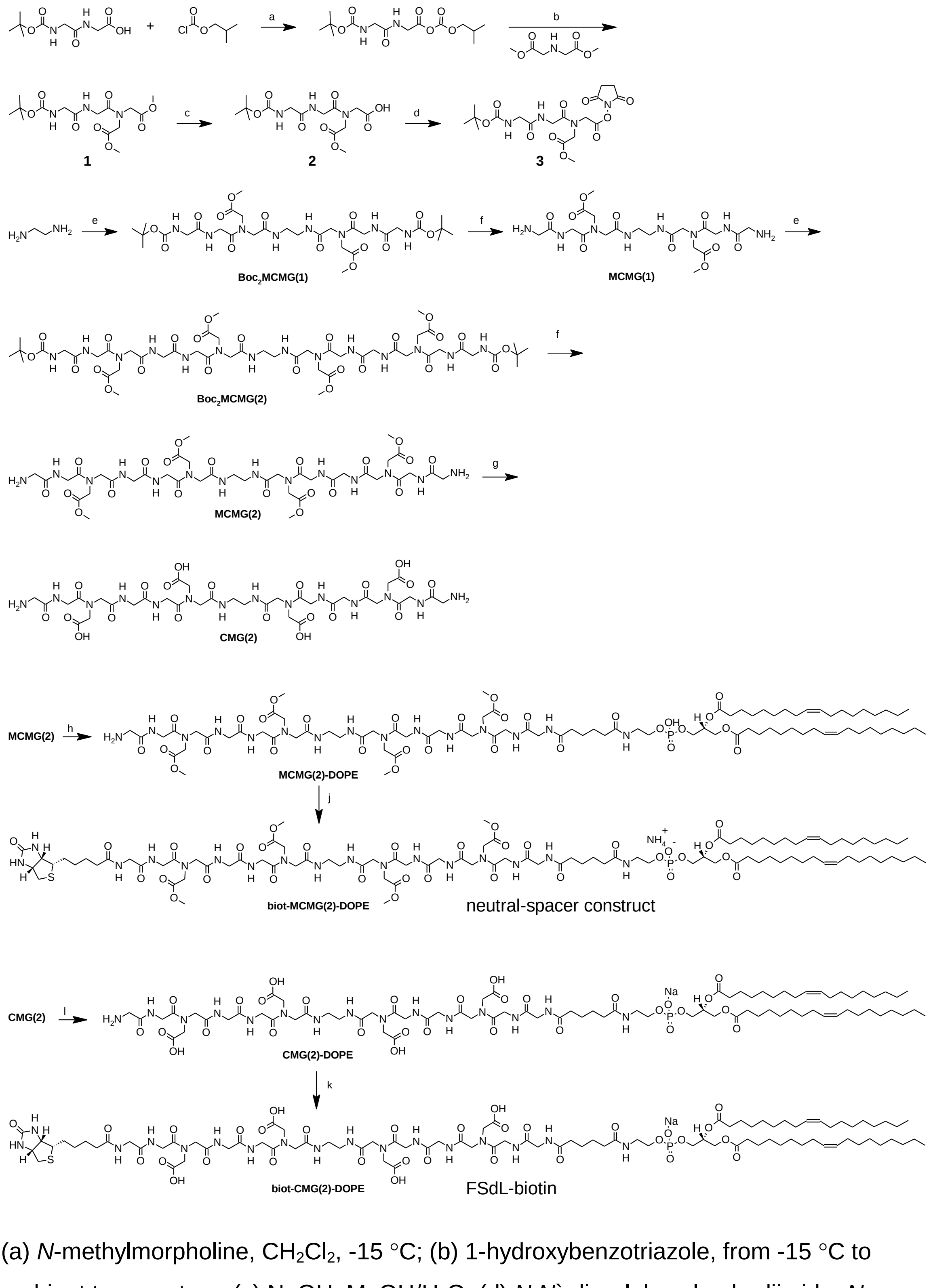

(a) *N*-methylmorpholine, CH_2Cl_2, -15 °C; (b) 1-hydroxybenzotriazole, from -15 °C to ambient temperature; (c) NaOH, MeOH/H_2O; (d) *N,N`*-dicyclohexylcarbodiimide, *N*-hydroxysuccinimide, DMF; (e) **3**, Et_3N, DMSO; (f) CF_3COOH; (g) Et_3N, H_2O; (h)

DOPE-Ad-ONSu in dichloroethane, Et_3N, pyridine; (i) DOPE-Ad-ONSu in dichloroethane, aq. $NaHCO_3$, *i*-PrOH/H_2O; (j) biotin *N*-oxysuccinimide ester, Et_3N, DMF; (k) biotin *N*-oxysuccinimide ester in DMF, aq. $NaHCO_3$, *i*-PrOH/H_2O.

Preparation of the tripeptide building block 3

Scheme 1a.1

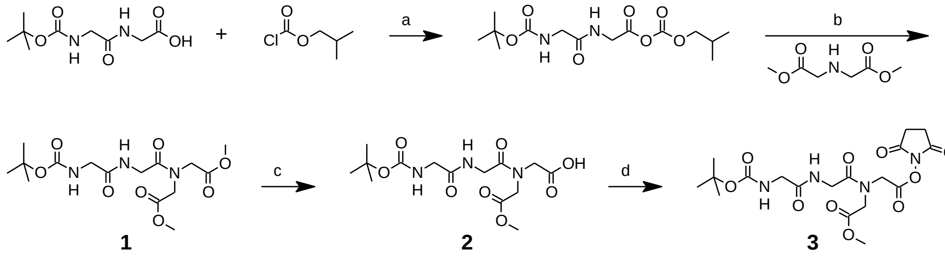

a) *N*-methylmorpholine, CH_2Cl_2, -15 °C; (b) 1-hydroxybenzotriazole, from -15 °C to ambient temperature; (c) NaOH, MeOH/H_2O; (d) *N,N`*-dicyclohexylcarbodiimide, *N*-hydroxysuccinimide, DMF.

Preparation of {[2-(2-tert-butoxycarbonylamino-acetylamino)-acetyl]-methoxycarbonylmethyl-amino}-acetic acid methyl ester (1)

N-Methylmorpholine (11.0 ml, 0.1 mol) was added to a stirred suspension of Boc-glycyl-glycine (23.2 g, 0.1 mol) in 150 ml methylene chloride, the solution was cooled to -15 °C and isobutyl chloroformate (13.7 g, 0.1 mol) was added for 10 min. Then 1-hydroxybenzotriazole and the solution of *N*-(carboxymethyl)glycine dimethyl ester (16.1 g, 0.1 mol) in 50 ml DMF were added to the reaction mixture at the same temperature. The resulting mixture was stirred for 30 min at 0 °C, then for 2 h at ambient temperature and evaporated to dryness. The residue was dissolved in 200 ml of methylene chloride and washed with 100 ml 0.5 M HCl and 200 ml 2% aq. $NaHCO_3$. Solvents were evaporated in vacuum and the residue was purified with column chromatography on silica gel (3% MeOH in $CHCl_3$) to give pure target compound (34.08 g, 91%) as a colourless glass.

TLC: R_f = 0.40 (5% MeOH in $CHCl_3$), R_f=0.49 (7:1 (v/v) chloroform/methanol). ^{1}H NMR (500 MHz, [D_6]DMSO, 30 ºC) δ, ppm: 7.826 (t, *J*=5.1 Hz, 1H; N$\underline{H}$CO), 6.979 (t, *J*=5.9 Hz, 1H; N$\underline{H}$COO), 4.348 and 4.095 (s, 2H; NC$\underline{H}_2$COO), 3.969 (d, *J*=5.1 Hz, 2H; COC$\underline{H}_2$NH), 3.689 and 3.621 (s, 3H; OC$\underline{H}_3$), 3.559 (d, *J*=5.9 Hz, 2H; COC$\underline{H}_2$NHCOO), 1.380 (s, 9H; $C(CH_3)_3$).

Preparation of {[2-(2-tert-butoxycarbonylamino-acetylamino)-acetyl]-methoxycarbonylmethyl-amino}-acetic acid (2)

0.2 M aqueous NaOH (325 ml) was added to a stirred solution of **1** (24.42 g, 65.05 mmol) in methanol (325 ml), reaction mixture was kept for 15 min at ambient temperature, acidified with acetic acid (5 ml) and evaporated to dryness. Column chromatography of the residue on silica gel (methanol – ethyl acetate 1:1) gave the target compound as mixture of free acid and Na-salt (20.44 g) which was dissolved in methanol/water/pyridine mixture (20:10:1, 350 ml) and passed through ion-exchange column (Dowex 50X4-400, pyridine form, 300 ml) to remove Na cations. Column was washed with the same mixture, eluate evaporated and dried in vacuum to give pure target compound (20.15 g, 86%) as a white solid.

TLC: R_f= 0.47 (*i*-PrOH / ethyl acetate/water 4:3:1), R_f= 0.34 (MeOH / ethyl acetate 1:1).

^{1}H NMR (500 MHz, [D_6]DMSO, 30 ºC), mixture of *cis*- and *trans*- conformers of peptide bond formed by *N*-carboxymethylglycine in ~ 3:1 ratio. Major conformer, δ, ppm: 7.717 (t, *J*=5 Hz, 1H; N<u>H</u>CO), 7.024 (t, *J*=5.9 Hz, 1H; N<u>H</u>COO), 4.051 (s, 2H; NC<u>H</u>$_2$COOCH$_3$), 3.928 (d, *J*=5 Hz, 2H; COC<u>H</u>$_2$NH), 3.786 (s, 2H; NC<u>H</u>$_2$COOH), 3.616 (s, 3H; OC<u>H</u>$_3$), 3.563 (d, *J*=5.9 Hz, 2H; COC<u>H</u>$_2$NHCOO), 1.381 (s, 9H; C(CH$_3$)$_3$); minor conformer, δ, ppm: 7.766 (t, *J*=5 Hz, 1H; N<u>H</u>CO), 7.015 (t, *J*=5.9 Hz, 1H; N<u>H</u>COO), 4.288 (s, 2H; NC<u>H</u>$_2$COOCH$_3$), 3.928 (d, *J*=5 Hz, 2H; COC<u>H</u>$_2$NH), 3.858 (s, 2H; NC<u>H</u>$_2$COOH), 3.676 (s, 3H; OC<u>H</u>$_3$), 3.563 (d, *J*=5.9 Hz, 2H; COC<u>H</u>$_2$NHCOO), 1.381 (s, 9H; C(CH$_3$)$_3$).

Preparation of {[2-(2-tert-Butoxycarbonylamino-acetylamino)-acetyl]-methoxycarbonylmethyl-amino}-acetic acid N-oxysuccinimide ester (3)

N,N'-Dicyclohexylcarbodiimide (15.83 g, 76.71 mmol) was added to an ice-cooled stirred solution of **2** (26.40 g, 73.06 mmol) and *N*-hydroxysuccinimide (8.83 g, 76.71 mmol) in DMF (210 ml). The mixture was stirred for 30 min at 0 °C then for 2 h at ambient temperature. Precipitated *N,N*'-dicyclohexylurea was filtered off and washed with DMF (80 ml). The filtrate and washings were evaporated in vacuum to a minimal volume, and the residue was stirred with

Et_2O (500 ml) for 1 h. Ether extract was decanted and the residue was dried in vacuum to give target compound as a white foam (32.57 g, 97%).

TLC: R_f = 0.71 (acetone/acetic acid 40:1), R_f = 0.43 (ethyl acetate / acetone/ water / acetic acid 50:10:1:1).

^{1}H NMR (500 MHz, DMSO[D_6], 30 ºC), mixture of *cis*- and *trans*- conformers of *N*-carboxymethylglycine unit *c*. 3:2. Major conformer; δ, ppm: 7.896 (t, *J*=5.1 Hz, 1H; N$\underline{H}$CO), 6.972 (t, *J*=5.9 Hz, 1H; N$\underline{H}$COO), 4.533 (s, 2H; NC$\underline{H}_2$COON), 4.399 (s, 2H; NC$\underline{H}_2$COOCH_3), 3.997 (d, *J*=5.1 Hz, 2H; COC$\underline{H}_2$NH), 3.695 (s, 3H; OC$\underline{H}_3$), 3.566 (d, *J*=5.9 Hz, 2H; COC$\underline{H}_2$NHCOO), 1.380 (s, 9H; C$(CH_3)_3$).

Minor conformer; δ, ppm: 7.882 (t, *J*=5.1 Hz, 1H; N$\underline{H}$CO), 6.963 (t, *J*=5.9 Hz, 1H; N$\underline{H}$COO), 4.924 (s, 2H; NC$\underline{H}_2$COON), 4.133 (s, 2H; NC$\underline{H}_2$COOCH_3), 4.034 (d, *J*=5.1 Hz, 2H; COC$\underline{H}_2$NH), 3.632 (s, 3H; OC$\underline{H}_3$), 3.572 (d, *J*=5.9 Hz, 2H; COC$\underline{H}_2$NHCOO), 1.380 (s, 9H; C$(CH_3)_3$).

Synthesis of MCMG(2) and CMG(2) diamines

Scheme 1a.2

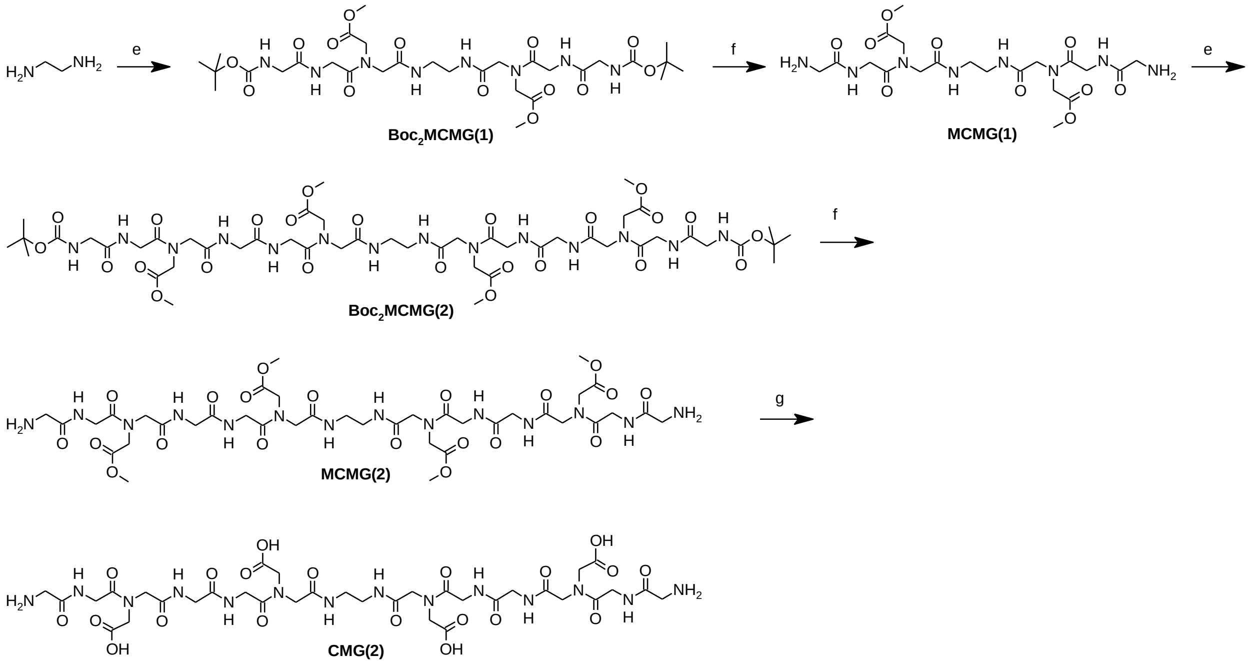

(e) **3**, Et_3N, DMSO; (f) CF_3COOH; (g) Et_3N, H_2O.

Preparation of Boc$_2$MCMG(1)

A solution of ethylenediamine (808 mg, 13.44 mmol) and Et_3N (1.87 ml, 13.5 mmol) in DMSO (5 ml) was added to a stirred solution of **3** (15.42 g, 33.64 mmol) in DMSO (50 ml). The reaction mixture was stirred for 30 min at ambient temperature and acidified with acetic acid (1.2 ml), then fractionated with Sephadex LH-20 column (column volume 1200 ml, eluent – MeOH/water 2:1 + 0.2% AcOH). Fractions containing Boc$_2$MCMG(1) were combined, solvents evaporated and the residue was concentrated in vacuum. The product was additionally purified by silica gel column chromatography using 2-propanol/ethyl acetate/water (2:6:1) as eluent. Fractions containing pure Boc$_2$MCMG(1) were combined, solvents evaporated and a residue was dried in vacuum to give target Boc$_2$MCMG(1) as colourless foam (8.41 g, 84 %).

TLC: R_f= 0.48 (*i*-PrOH/ ethyl acetate/water 2:3:1).

^{1}H NMR (500 MHz, [D$_6$]DMSO, 30°C), mixture of conformers ~3:2: 8.166, 8.125, 7.917 and 7.895 (m, total 2H; 2 $CON\underline{H}CH_2$), 7.793 (m, 2H; $N\underline{H}CH_2CH_2N\underline{H}$), 7.001 (br. t, 2H; 2 NHCOO), 4.277-3.893 (total 12H; 2 CH_2COO, 4 NCH_2CO), 3.690 and 3.635 (s, total 6H; 2 $COOCH_3$), 3.567 (d, *J*=5.8 Hz, 4H; 2 $C\underline{H}_2NHCOO$), 3.131 (m, 4H; $NHC\underline{H}_2C\underline{H}_2NH$), 1.379 (s, 18H; 2 $C(CH_3)_3$) ppm.

MS (MALDI-TOF): calc. for $C_{30}H_{50}N_8O_{14}$ $Mw_{isotopic}$=746, found 769 [M+Na], 785 [M+K].

Preparation of MCMG(1)

Trifluoroacetic acid (25 ml) was added to a stirred solution of Boc$_2$MCMG(1) (4.88 g, 6.535 mmol) in methylene chloride (25 ml) and the solution was kept for 1 h at ambient temperature. Then a reaction mixture was concentrated and the residue was evaporated three times with anhydrous MeOH (50 ml), then a residue was extracted three times with Et_2O (100 ml) to remove traces of trifluoroacetic acid. The resulted precipitate (as a white solid) was dried to give 5.06 g (~100 %) of MCMG(1) as bis-trifluoroacetic salt.

TLC: R_f= 0.23 (ethanol/water/pyridine/acetic acid 5:1:1:1).

^{1}H NMR (500 MHz, D_2O, 30°C), mixture of conformers (~5:4): 4.400-4.098 (total 12H; 2 CH_2COO, 4 NCH_2CO), 3.917 (s, 4H; 2 $COC\underline{H}_2NH_2$), 3.829 and 3.781 (s, total 6H; 2 $COOCH_3$), 3.394 (m, 4H; $NHC\underline{H}_2C\underline{H}_2NH$) ppm.
MS (MALDI-TOF): calc. for $C_{20}H_{34}N_8O_{10}$ $Mw_{isotopic}$=546, found 547 [M+H], 569 [M+Na], 585 [M+K].

Preparation of Boc$_2$MCMG(2)

A solution of **3** (7.49 g, 16.34 mmol) in DMSO (17 ml) and Et_3N (2.73 ml, 19.6 mmol) was added to the stirred solution of MCMG(1) bis-trifluoroacetic salt (5.06 g, 6.535 mmol) in DMSO (13 ml). The reaction mixture after stirring for 2 h at ambient temperature was acidified with acetic acid (4.0 ml) and fractionated with Sephadex LH-20 column chromatography (column volume 1200 ml, eluent – MeOH/water 2:1 + 0.2% AcOH). Fractions containing pure Boc_2MCMG(2) were combined, solvents evaporated and the residue was dried in vacuum to give target Boc_2MCMG(2) as colourless foam (7.82 g, 97 %).
TLC: R_f= 0.25 (^{i}PrOH/ ethyl acetate/water 2:3:1).
^{1}H NMR (500 MHz, [D_6]DMSO, 30°C), mixture of conformers: 8.393-7.887 (total 6H; 6 $CON\underline{H}CH_2$), 7.775 (m, 2H; $N\underline{H}CH_2CH_2N\underline{H}$), 6.996 (br. t, 2H; 2 NHCOO), 4.299-3.730 (total 28H; 4 CH_2COO, 10 NCH_2CO), 3.691 and 3.633 (s, total 12H; 4 $COOCH_3$), 3.564 (d, J=5.8 Hz, 4H; 2 $C\underline{H}_2NHCOO$), 3.129 (m, 4H; $NHC\underline{H}_2C\underline{H}_2NH$), 1.380 (s, 18H; 2 $C(CH_3)_3$) ppm.
MS (MALDI-TOF): calc. for $C_{48}H_{76}N_{14}O_{24}$ $Mw_{isotopic}$=1233, found 1256 [M+Na], 1272 [M+K].

Preparation of MCMG(2) diamine

Boc_2MCMG(2) (1212 mg, 0.983 mmol) was dissolved in CF_3COOH (4 ml) and the solution was kept for 30 min at r.t. Trifluoroacetic acid was evaporated in vacuum and the residue was extracted three times with Et_2O (trituration with 25 ml of Et_2O followed by filtration) to remove residual CF_3COOH, and the obtained white powder was dried in vacuum. Yield of MCMG(2) bis-trifluoroacetic salt was 1238 mg (~100 %). The powder was dissolved in 8 mL of water and then was freeze-dried. Weight of freeze-dried MCMG(2) bis-

trifluoroacetic salt was larger than theoretical by 10% due to stability of hydrates.
TLC: R_f = 0.21 (ethanol/water/pyridine/acetic acid 5:1:1:1).
^{1}H NMR (500 MHz, $[D_2]H_2O$, 30°C), mixture of conformers: 4.430-4.014 (total 28H; 4 CH_2COO, 10 NCH_2CO), 3.911 (s, 4H; 2 $COC\underline{H}_2NH_2$), 3.823 and 3.772 (s, total 12H; 4 $COOCH_3$), 3.386 (m, 4H; $NHC\underline{H}_2C\underline{H}_2NH$) ppm.
MS (MALDI-TOF): calc. for $C_{38}H_{60}N_{14}O_{20}$ $Mw_{isotopic}$=1032, found 1033 [M+H], 1055 [M+Na].

Preparation of CMG(2) diamine

To the solution of MCMG(2) bis-trifluoroacetic salt (618 mg, 0.49 mmol) in water (20 mL) Et_3N (0.5 mL, 3.6 mmol) was added, and the solution was kept for 15 h at r.t.. The reaction mixture was evaporated to dryness, the residue dissolved in water (3 ml) and the solution was desalted on Sephadex LH-20 column (column volume 250 mL, eluent - MeOH/water 1:1 + 0.05 M pyridine acetate). Fractions, containing pure CMG(2), were evaporated to ~4 ml volume and freeze dried. Yield of CMG(2) diamine (internal salt) was 431 mg (90%).
For large-scale syntheses CMG(2) diamine may be used without desalting on Sephadex, freeze-drying of the residue after evaporation of the reaction mixture gave di-TFA,di-Et_3N salt with some excess of Et_3N (0.35 M/M by NMR data) in quantitative yield.
TLC: R_f = 0.50 (^{i}PrOH/MeOH/acetonitrile/water 4:3:3:4 + 3% conc. aq. NH_3), R_f = 0.43 (MeOH/$CHCl_3$/water 2:1:1), R_f = 0.19 (MeOH/$CHCl_3$/water 3:1:1).
^{1}H NMR of CMG(2) internal salt (500 MHz, $[D_2]H_2O$, 30°C), mixture of conformers: 4.328-4.006 (total 28H; 4 CH_2COO, 10 NCH_2CO), 3.907 (s, 4H; 2 $COC\underline{H}_2NH_2$), 3.381 (m, 4H; $NHC\underline{H}_2C\underline{H}_2NH$) ppm.
MS (MALDI-TOF): calc. for $C_{34}H_{52}N_{14}O_{20}$ $Mw_{isotopic}$=976, found 977 [M+H], 999 [M+Na], 1015 [M+K].

Synthesis of MCMG(2) and CMG(2) DOPE derivatives

Scheme 1a.3

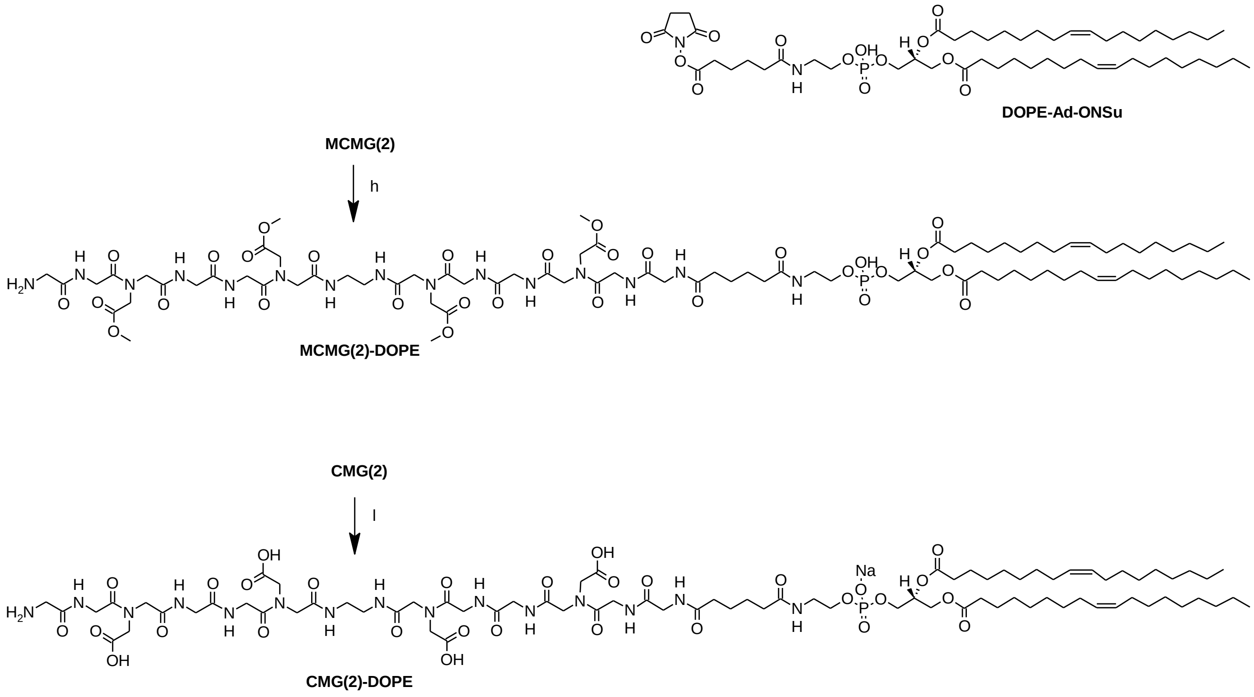

(h) DOPE-Ad-ONSu in dichloroethane, Et_3N, pyridine; (i) DOPE-Ad-ONSu in dichloroethane, aq. $NaHCO_3$, *i*-PrOH/H_2O.

Preparation of DOPE-Ad-ONSu[1]

To a solution of *bis*(N-hydroxysuccinimidyl) adipate (700 mg, 2.05 mmol) in dry *N*,*N*-dimethyl formamide (30 ml) were added 1,2-O-dioleoyl-*sn*-glycero-3-phosphatidylethanolamine (DOPE) (300 mg, 0.403 mmol) in chloroform (15 ml) and triethylamine (50 μl). The mixture was stirred for 2 h at room temperature, then neutralized with acetic acid and concentrated in *vacuo*. Column chromatography on Sephadex LH-20 (chloroform/methanol 1:1, 0.2% acetic acid) of the residue yielded the activated lipid (370 mg, 95%) as a colorless syrup.

TLC: R_f = 0.50 (chloroform/methanol/water 12:6:1).

^{1}H NMR (500 MHz, $[D_3]CHCl_3/[D_4]CH_3OH$ 1:1, 30°C): 5.50 (m, 4H, 2×(-C<u>H</u>=C<u>H</u>-), 5.39 (m, 1H, -OCH_2-C<u>H</u>O-CH_2O-), 4.58 (dd, 1H, J=3.67, J=11.98, -CCOOHC<u>H</u>-CHO-CH_2O-), 4.34 (dd, 1H, J=6.61, J=11.98, -CCOO<u>H</u>CH-CHO-CH_2O-), 4.26 (m, 2H, PO-C<u>H</u>$_2$-CH_2-NH_2), 4.18 (m, 2H, -C<u>H</u>$_2$-OP), 3,62 (m, 2H, PO-CH_2-C<u>H</u>$_2$-NH_2), 3.00 (s, 4H, ONSuc), 2.80 (m, 2H, -C<u>H</u>$_2$-CO (Ad), 2.50 (m,

4H, 2×(-C$\underline{H}_2$-CO), 2.42 (m, 2H, -C$\underline{H}_2$-CO (Ad), 2.17 (m, 8H, 2×(-C$\underline{H}_2$-CH=CH-C$\underline{H}_2$-), 1.93 (m, 4H, $COCH_2C\underline{H}_2C\underline{H}_2CH_2CO$), 1.78 (m, 4H, 2×($COCH_2C\underline{H}_2$-), 1,43, 1.47 (2 bs, 40H, 20 CH_2), 1.04 (~t, J≈7 Hz, 6H; 2 CH_3)

Preparation of MCMG(2)-Ad-DOPE amine[1]

To the intensively stirred solution of MCMG(2) (hydrated, 10% of H_2O, 378 mg, 0.270 mmol) in pyridine (5 mL) a solution of DOPE-Ad-ONSu (174 mg, 0.180 mmol) in dichloroethane (0.87 mL) and Et_3N (0.1 mL, 0.72 mmol) were added. The reaction mixture was stirred for 2 h and then acidified with 0.2 mL of AcOH and evaporated to minimal volume at 35ºC. The residue was dried in vacuum (solid foam). The obtained mixture was separated on silica gel column (2.8 x 33 cm, ~ 200 mL of silica gel in $CHCl_3$/MeOH 5:1). The mixture was placed on column in $CHCl_3$/MeOH (5:1) and the components were eluted with $CHCl_3$/MeOH (1:1) and then with alteration of MeOH/$CHCl_3$/water composition from 7:7:1 to 6:6:1. First eluted was DOPE-Ad-MCMG(2)-Ad-DOPE (R_f = 0.72, MeOH/$CHCl_3$/water 6:6:1), second eluted was aimed MCMG(2)-Ad-DOPE amine (R_f = 0.34, MeOH/$CHCl_3$/water 6:6:1). Remaining MCMG(2) diamine was eluted with MeOH/1 M Py·HOAc (3:2, R_f = 0.47), recovery was 129 mg (42%) as MCMG(2) diacetate. Fractions, containing pure MCMG(2)-Ad-DOPE amine were combined, evaporated and dried in vacuum. The residue was dissolved in 5% AcOH and freeze-dried. Yield of MCMG(2)-Ad-DOPE amine was 132 mg (26% on loaded or 45% on used up MCMG(2)).

^{1}H NMR (500 MHz, $[D_3]CHCl_3/[D_4]CH_3OH$ 1:2, 30°C): 5.507 (m, 4H; 2 $CH_2C\underline{H}=C\underline{H}CH_2$), 5.406 (m, 1H; $OCH_2C\underline{H}CH_2O$), 4.605 (dd, J_{gem}=12.1 Hz, 1H; $OC\underline{H}CHCH_2O$), 4.510-3.849 (total 51H; 4 CH_2COO, 12 NCH_2CO, $OC\underline{H}CHC\underline{H}_2O$, OCH_2CH_2N, 4$CH_2COOC\underline{H}_3$), 3.586 (m, 4H; $NHC\underline{H}_2C\underline{H}_2NH$), 2.491 and 2.419 (m, total 8H; 4 CH_2CO), 2.194 (m, 8H; 2 $C\underline{H}_2CH=CHC\underline{H}_2$), 1.837 and 1.784 (m, total 8H; 4 $C\underline{H}_2CH_2CO$), 1.478 (m, 40H; 20 CH_2), 1.061 (~t, J≈7 Hz, 6H; 2 CH_3) ppm.

MS (MALDI-TOF): calc. for $C_{85}H_{144}N_{15}O_{30}P$ $Mw_{isotopic}$=1886, found 1887 [M+H], 1909 [M+Na], 1925 [M+K].

Preparation of CMG(2)-Ad-DOPE amine[1]

To the intensively stirred solution of CMG(2) diamine (425 mg, 0.435 mmol of internal salt) in *i*-PrOH/water mixture (*i*-PrOH/water 3:2, 10 mL) a solution of DOPE-Ad-ONSu (211 mg, 0.218 mmol) in dichloroethane (0.4 mL) and 1 M aq. solution of $NaHCO_3$ (0.435 mL, 0.435 mmol) were added. The reaction mixture was stirred for 2 h and then acidified with 0.2 mL of AcOH and evaporated to minimal volume at 35ºC. The solid residue was dried in vacuum (solid foam) and then thoroughly extracted with $CHCl_3$/MeOH mixture ($CHCl_3$/MeOH 4:1, several times with 10 mL, TLC control). The extracted residue consisted of unreacted CMG(2) and salts (~ 50% of CMG(2) was recovered by desalting of combined the residue and a fractions after chromatography on silica gel according to procedure described in the CMG(2) synthesis.). The combined $CHCl_3$/MeOH extracts (solution of CMG(2)-Ad-DOPE amine, DOPE-Ad-CMG(2)-Ad-DOPE, N-oxysuccinimide and some CMG(2)) were evaporated in vacuum and dried. The obtained mixture was separated on silica gel column (2.8 x 33 cm, ~ 200 mL of silica gel in $CHCl_3$/MeOH 5:1). The mixture was placed on column in MeOH/$CHCl_3$/water mixture (MeOH/$CHCl_3$/water 6:3:1 + 0.5% of pyridine) and the components were eluted in a stepwise ternary gradient: MeOH/$CHCl_3$/water composition from 6:3:1 to 6:2:1 and then to 6:2:2 (all with 0.5% of pyridine). DOPE-Ad-CMG(2)-Ad-DOPE was eluted first (R_f = 0.75, MeOH/$CHCl_3$/water 3:1:1), followed by desired DOPE-Ad-CMG(2)amine (R_f = 0.63, MeOH/$CHCl_3$/water 3:1:1), last eluted was CMG(2) (R_f = 0.31, MeOH/$CHCl_3$/water 3:1:1). Fractions, containing pure CMG(2)-Ad-DOPE amine were combined and evaporated to dryness. To remove any low molecular weight impurities and solubilised silica gel the residue was dissolved in ^{i}PrOH/water 1:2 mixture (2 mL), and was passed through Sephadex LH-20 column (column volume 130 mL, eluent - ^{i}PrOH/water 1:2 + 0.25% of pyridine). Fractions containing pure CMG(2)-Ad-DOPE amine were combined and evaporated (~ 20% of 2-propanol was added to prevent foaming) to dryness, the residue was dissolved in water (~4 mL) and freeze-dried. Yield of CMG(2)-Ad-DOPE amine was 270 mg (68% on DOPE-Ad-ONSu, 34% on loaded or 68% on used up CMG(2) diamine).

^{1}H NMR (500 MHz, $[D_2]H_2O/[D_4]CH_3OH$ 2:1, 30°C): 5.505 (m, 4H; 2 $CH_2C\underline{H}{=}C\underline{H}CH_2$), 5.476 (m, 1H; $OCH_2C\underline{H}CH_2O$), 4.626 (dd, J_{gem}=11.6 Hz, 1H; $OC\underline{H}CHCH_2O$), 4.461-4.084 (total 37H; 4 CH_2COO, 11 NCH_2CO, $OC\underline{H}CHC\underline{H}_2O$, OCH_2CH_2N), 4.002 (s, 2H; $COC\underline{H}_2NH_2$), 3.573 (m, 4H; $NHC\underline{H}_2C\underline{H}_2NH$), 2.536-2.463 (m, total 8H; 4 CH_2CO), 2.197 (m, 8H; 2 $C\underline{H}_2CH{=}CHC\underline{H}_2$), 1.807 (m, 8H; 4 $C\underline{H}_2CH_2CO$), 1.480 (m, 40H; 20 CH_2), 1.063 (~t, J≈6 Hz, 6H; 2 CH_3) ppm.

MS (MALDI-TOF): calc. for $C_{81}H_{136}N_{15}O_{30}P$ $Mw_{isotopic}$=1830, found 1831 [M+H], 1853 [M+Na], 1869 [M+K].

Synthesis of biotin-MCMG(2) and biotin-CMG(2) DOPE derivatives

Scheme 1a.4

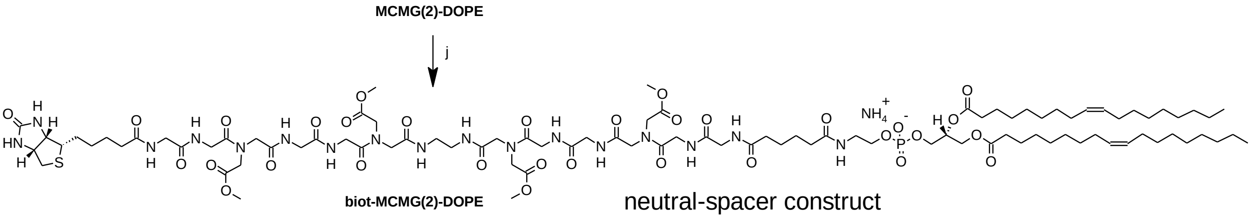

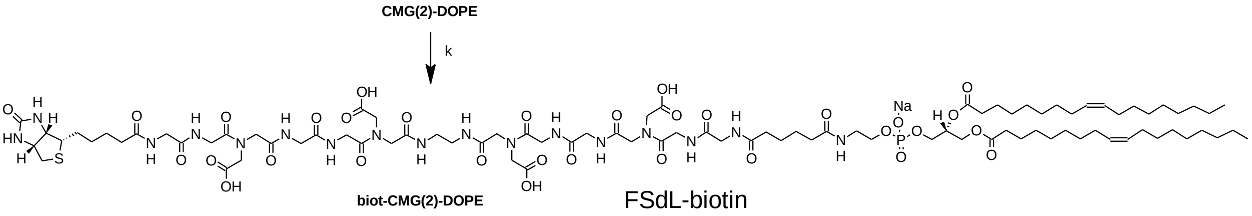

(j) biotin *N*-oxysuccinimide ester, Et_3N, DMF; (k) biotin *N*-oxysuccinimide ester in DMF, aq. $NaHCO_3$, *i*-PrOH/H_2O.

Preparation of biot-MCMG(2)-DOPE (neutral-spacer construct)

To the stirred solution of MCMG(2)-Ad-DOPE amine (9.7 mg, 5.14 micromole) in DMF (0.6 mL) the solution of biotin N-oxysuccinimide ester (2.3 mg, 6.74 micromole) in DMF (50 μL) and Et_3N (1.7 μL, 12.2 micromole) were added. The reaction mixture was stirred for 3 h at r.t. and then acidified with AcOH (20 μL). The resulted solution was passed through Sephadex LH-20 column (80 mL, eluent – i-PrOH/water 1:2 + 0.5% of pyridine and 0.25% of AcOH).

Fractions, containing pure biotin-MCMG(2)-Ad-DOPE were evaporated and dried in vacuum. The residue was dissolved i-PrOH/water (1:4 by volume), aqueous 1 M NH_3 was added (10 μL) and the solution was freeze-dried. Yield of biotin-MCMG(2)-Ad-DOPE (ammonium salt) was 10.3 mg (94%).

TLC: R_f = 0.57 ($CHCl_3$/methanol/water 6:6:1 by volume).

^{1}H NMR (700 MHz, $[D_4]CH_3OH/[D_2]H_2O/CDCl_3$ 4:1:1, 30°C) δ 5.523 (m, 4H; 2 $CH_2C\underline{H}=C\underline{H}CH_2$), 5.430 (m, 1H; $OCH_2C\underline{H}CH_2O$), 4.717 (dd, J = 7.6, 5.1 Hz, 1H; $NHC\underline{H}$ of biotin), 4.627 (dd, J = 12.1, 2.7 Hz, 1H; $OC\underline{H}CHCH_2O$), 4.566-3.854 (m, total 52H; $NHC\underline{H}$ of biotin, 4 CH_2COO, 12 NCH_2CO, $OC\underline{H}CHC\underline{H}_2O$, OCH_2CH_2N, 4 OCH_3), 3.553 (m, 4H; $NHC\underline{H}_2C\underline{H}_2NH$), 3.412 (m, 1H; $NHCHC\underline{H}$ of biotin), 3.13 (dd, J = 12.8, 5.1 Hz, 1H; $NHCHC\underline{H}$ of biotin), 2.919 (d, J = 12.8 Hz, 1H; $NHCHC\underline{H}$ of biotin), 2.548-2.423 (m, total 10H; 5 CH_2CO), 2.205 (m, 8H; 2 $C\underline{H}_2CH=CHC\underline{H}_2$), 1.960-1.750 (m, 12H; $C\underline{H}_2CH_2CH_2CH_2CO$ of biotin, 5 $C\underline{H}_2CH_2CO$), 1.651 (m, 2H; $CH_2C\underline{H}_2CH_2CH_2CO$), 1.504 (m, 40H; 20 CH_2), 1.068 (t, J = 7.0 Hz, 6H; 2 CH_3).

MS (MALDI-TOF): calc. for $C_{95}H_{158}N_{17}O_{32}PS$ $Mw_{isotopic}$=2112, found 2113 [M+H], 2135 [M+Na], 2151 [M+K].

Insoluble in water, soluble in water/2-propanol mixtures.

Preparation of biot-CMG(2)-DOPE (FSdL-biotin)[1]

To the intensively stirred solution of CMG(2)-Ad-DOPE amine (366 mg, 0.200 mmol) in ^{i}PrOH/water mixture (^{i}PrOH/water 1:1, 15 mL) 1 M aq. solution of $NaHCO_3$ (1.2 mL, 1.2 mmol) and then the solution of biotin N-oxysuccinimide ester (75.1 mg, 0.220 mmol) in DMF (1.5 mL) were added. The reaction mixture was stirred for 2.5 h and then acidified with 0.15 mL of AcOH and evaporated to minimal volume (beginning of a precipitate formation) at 35ºC. The residue was diluted with water (1.5 ml) and 0.05 ml of pyridine was added. The resulted solution was passed through Sephadex LH-20 column (column volume 300 mL, eluent - ^{i}PrOH/water 1:2 + 0.5% of pyridine and 0.25% of AcOH). Fractions, containing pure biotin-CMG(2)-Ad-DOPE were combined and evaporated (~ 20% of 2-propanol was added to prevent foaming) to dryness, the residue was dissolved in water (~ 4 mL, containing 0.05 ml of pyridine) and freeze-dried. Yield of biotin-CMG(2)-Ad-DOPE (pyridinium salt)

was 384.5 mg (~0.180 mmol). The product was dissolved in water (3 ml) containing $NaHCO_3$ (0.18 ml of 1 M aqueous solution, 0.180 mmol) and freeze-dried. Yield of biotin-CMG(2)-Ad-DOPE (sodium salt) was 374.3 mg (91% on CMG(2)-Ad-DOPE amine).

TLC: R_f = 0.64 (MeOH/$CHCl_3$/water 6:2:1.

^{1}H NMR (600 MHz, $[D_2]H_2O/[D_4]CH_3OH$ 2:1, 30°C): 5.508 (m, 4H; 2 $CH_2C\underline{H}{=}C\underline{H}CH_2$), 5.458 (m, 1H; $OCH_2C\underline{H}CH_2O$), 4.766 (dd, J=7.9 Hz, J=4.9 Hz, 1H; NHC$\underline{H}$ of biotin), 4.619 (dd, J_{gem}=11.6 Hz, 1H; $OC\underline{H}CHCH_2O$), 4.584 (dd, J=7.9 Hz, J=4.5 Hz, 1H; NHC$\underline{H}$ of biotin), 4.470-4.078 (total 39H; 4 CH_2COO, 12 NCH_2CO, $OC\underline{H}CHC\underline{H}_2O$, OCH_2CH_2N), 3.571 (m, 4H; $NHC\underline{H}_2C\underline{H}_2NH$), 3.483 (m, 1H; NHCHC$\underline{H}$ of biotin), 3.162 (dd, J=13 Hz, J=4.9 Hz, 1H; NHCHC$\underline{H}$ of biotin), 2.941 (dd, J=13 Hz, J‹2 Hz, 1H; NHCHC$\underline{H}$ of biotin), 2.527-2.458 (m, total 10H; 5 CH_2CO), 2.196 (m, 8H; 2 $C\underline{H}_2CH{=}CHC\underline{H}_2$), 1.885 (m, 2H; $C\underline{H}_2CH_2CH_2CH_2CO$ of biotin), 1.806 (m, 10H; 5 $C\underline{H}_2CH_2CO$), 1.627 (m, 2H; $CH_2C\underline{H}_2CH_2CH_2CO$), 1.480 (m, 40H; 20 CH_2), 1.069 (~t, J≈7 Hz, 6H; 2 CH_3) ppm.

MS (MALDI-TOF): calc. for $C_{91}H_{150}N_{17}O_{32}PS$ $Mw_{isotopic}$=2056, found 2079 [M+Na], 2095 [M+K], 2101 [MNa+Na].

Supplementary Note 1b. **Synthetic scheme and detailed protocol for synthesis of FScL-biotin.**

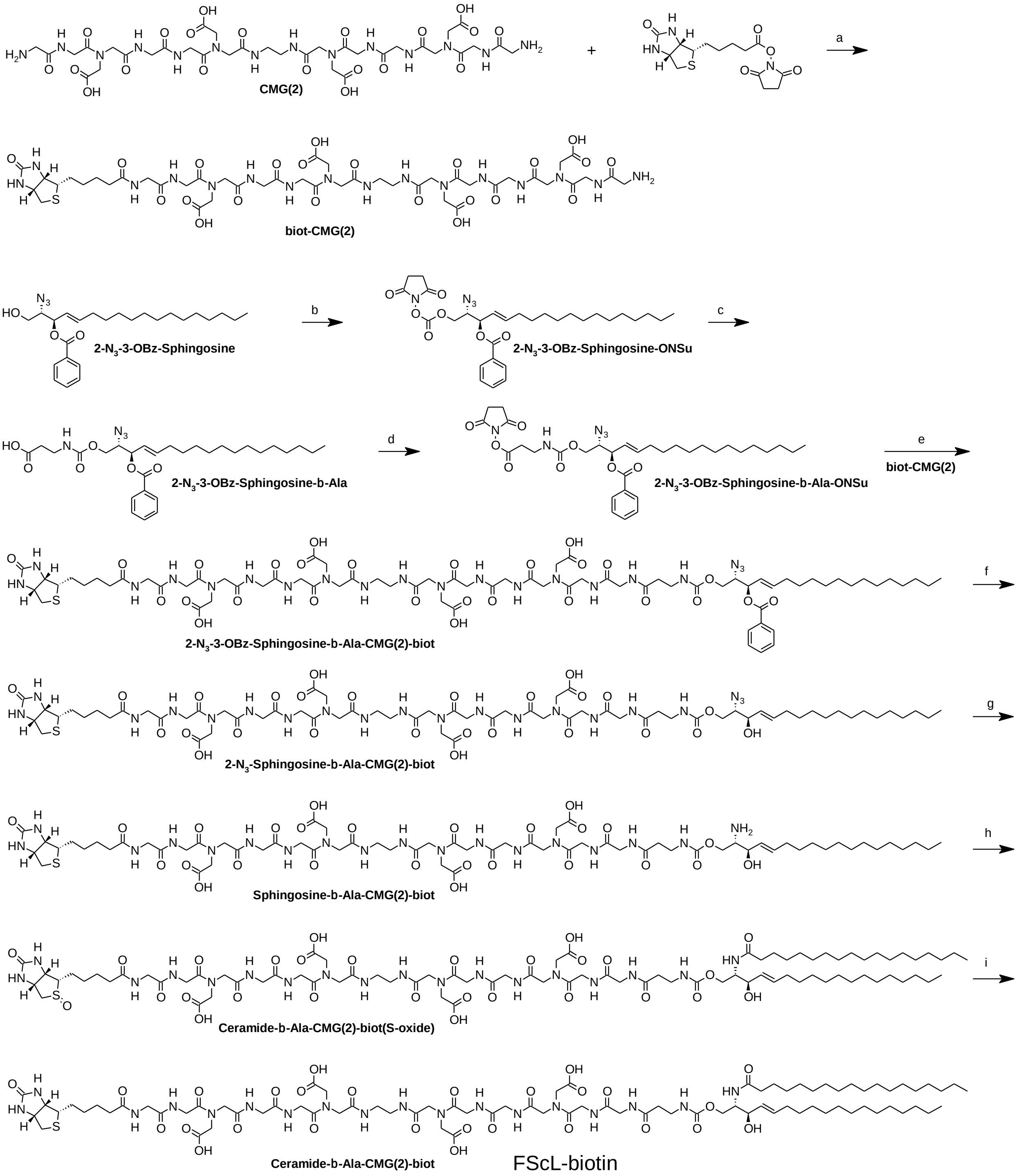

(a) biotin *N*-oxysuccinimide ester in DMF, aq. $NaHCO_3$, *i*-PrOH/H_2O; (b) disuccinimidylcarbonate, Et_3N, DMF/CH_2Cl_2 (c) β-alanine, Et_3N, DMSO/DMF/CH_2Cl_2; (d) disuccinimidylcarbonate, Et_3N, DMF/1,2-dichloroethane; (e) biot-CMG(2) amine, aq. $NaHCO_3$, 1,2-dichloroethane/DMSO/H_2O; (f) Et_3N, H_2O/MeOH; (g) dithiothreitol,

Et_3N, H_2O/MeOH; (h) Stearoyl-ONSu in 1,2-dichloroethane, aq. $NaHCO_3$, H_2O/2-propanol; (i) N-Methyl-mercaptoacetamide. H_2O, 69 h at 40 °C.

Preparation of biotin-CMG(2) amine

Scheme 1b.1

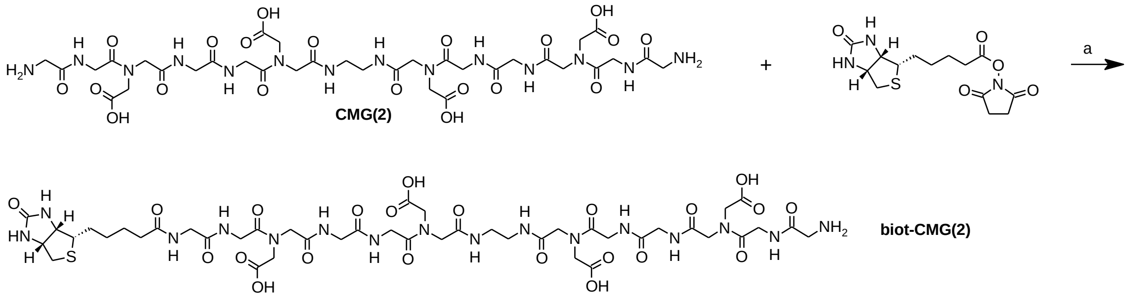

(a) biotin *N*-oxysuccinimide ester in DMF, aq. $NaHCO_3$, *i*-PrOH/H_2O.

Preparation of biot-CMG(2) amine

To a thoroughly stirred solution of CMG(2) diamine internal salt (693 mg, 0.709 mM; described in Supplementary Note 1) in a mixture of 2-propanol/water (1:1 by volume, 20 mL) a solution of biot-ONSu (242 mg, 0.709 mM) in DMF (4.84 mL) and 1 M aqueous $NaHCO_3$ (2.84 mL) were added, and the mixture was stirred for 30 min at ambient temperature. The reaction mixture was acidified with AcOH (122 μL), evaporated and dried. The obtained mixture was separated on silica gel column (~ 350 mL of silica gel in $CHCl_3$/MeOH 4:1). The mixture was placed on column in MeOH/$CHCl_3$/water mixture (1:4:3 by volume, 4 mL) and the components were eluted with MeOH/$CHCl_3$/water (1:3:1 by volume). Di-biotin derivative biot-CMG(2)-biot was eluted first (TLC: R_f = 0.48, MeOH/$CHCl_3$/water 3:1:1), followed by desired biot-CMG(2) amine (TLC: R_f = 0.30, MeOH/$CHCl_3$/water 3:1:1), last eluted was CMG(2) diamine (TLC: R_f = 0.19, MeOH/$CHCl_3$/water 3:1:1). Fractions, containing pure biot-CMG(2) amine were combined and evaporated to dryness. To remove any low molecular weight impurities and solubilised silica gel the residue was dissolved in water (2 mL), and was passed through Sephadex LH-20 column (column volume 170 mL, eluent - MeOH/water 1:3 by volume). After evaporating a residue was dissolved in water (~4 mL) and freeze-dried. Yield of biot-CMG(2) amine tri-Na salt was 316 mg (35% on loaded or 51% on used up CMG(2) diamine). Yield of biot-CMG(2)-biot was 190 mg (18% on CMG(2) diamine) and recovery of CMG(2) diamine was 231 mg (32%).

^{1}H NMR of biot-CMG(2) amine (700 MHz, $[D_2]H_2O$, 30°C): δ 4.637 (dd, *J* = 7.9, 4.6 Hz, 1H; NHC<u>H</u> of biotin), 4.458 (dd, *J* = 7.9, 4.5 Hz, 1H; NHC<u>H</u> of biotin), 4.321-3.900 (total 32H; 4 CH_2COO, 12 NCH_2CO), 3.438-3.337 (m, 5H; NCH_2CH_2N and NHCHC<u>H</u> of biotin), 3.030 (dd, *J* = 13.0, 5.0 Hz, 1H; NHCHC<u>H</u> of biotin), 2.812 (d, *J* = 13.0 Hz, 1H; NHCHC<u>H</u> of biotin), 2.384 (~t, *J* = 7.4 Hz, 2H; $COCH_2$ of biotin), 1.761, (m, 1H; $COCH_2CH_2CH_2C$<u>H</u> of biotin), 1.697 (m, 2H; $COCH_2C$<u>H</u>$_2CH_2CH_2$ of biotin), 1.621 (m, 1H; $COCH_2CH_2CH_2C$<u>H</u> of biotin), 1.467 (m, 2H; $COCH_2CH_2C$<u>H</u>$_2CH_2$ of biotin) ppm.
MS (MALDI-TOF): calc. for $C_{44}H_{66}N_{16}O_{22}S$ $Mw_{isotopic}$=1202, found 1203 [M+H], 1225 [M+Na].

Preparation of sphingosine-β-Ala-CMG(2)-biot

Scheme 1b.2

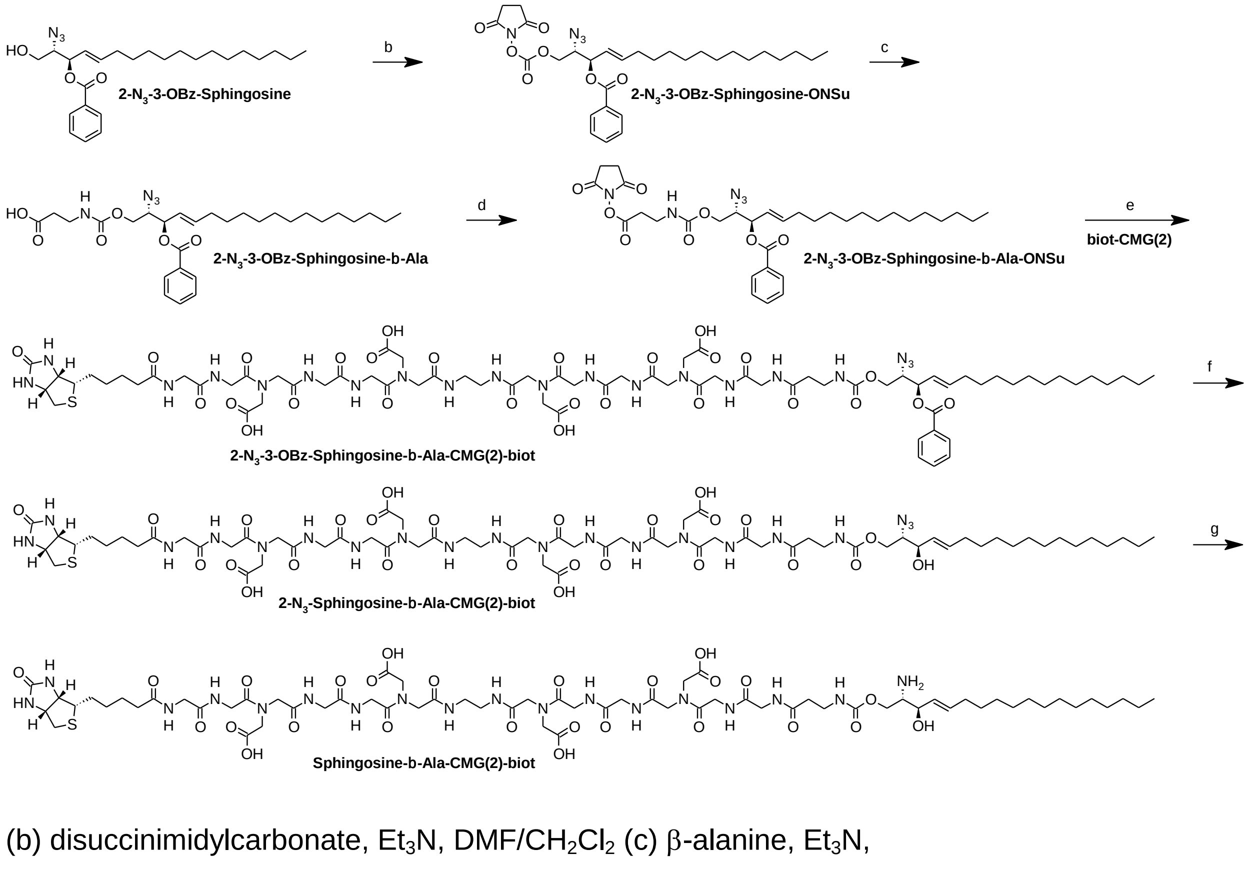

(b) disuccinimidylcarbonate, Et_3N, DMF/CH_2Cl_2 (c) β-alanine, Et_3N, $DMSO/DMF/CH_2Cl_2$; (d) disuccinimidylcarbonate, Et_3N, DMF/1,2-dichloroethane; (e) biot-CMG(2) amine, aq. $NaHCO_3$, 1,2-dichloroethane/DMSO/H_2O; (f) Et_3N, H_2O/MeOH; (g) dithiothreitol, Et_3N, H_2O/MeOH.

2-Azido-3-benzoyl-sphingosine-ONSu

To a stirred solution of (2S,3R,4E)-2-azido-3-benzoyloxy-octadec-4-ene-1-ol (2-azido-3-benzoyl-sphingosine[2]) (103 mg, 0.240 mmol) in a mixture of CH_2Cl_2 (3 mL) and dimethyl formamide (2 mL) disuccinimidylcarbonate (123 mg, 0.480 mmol) and Et_3N (33 μL, 0.24 mmol) were added, and the mixture was stirred for 20 h at ambient temperature. The reaction mixture was evaporated in vacuum (oil pump), the residue was dissolved in $CHCl_3$ and extracted with water (3 x 4 mL). Chloroform extract was evaporated and the residue was thoroughly dried in vacuum. Yield of 2-azido-3-benzoyl-sphingosine-ONSu was 130 mg (95%), white solid.

TLC: 2-azido-3-benzoyl-sphingosine R_f = 0.42, 2-Azido-3-benzoyl-Sphingosine-ONSu R_f = 0.32 (hexane/$CHCl_3$/2-propanol 15:5:1 by volume).

2-Azido-3-benzoyl-sphingosine-β-Ala

To a stirred solution of 2-azido-3-benzoyl-sphingosine-ONSu (130 mg, 0.228 mmol) in a mixture of CH_2Cl_2 (2 mL) and dimethyl formamide (2 mL) a solution of β-alanine (40.5 mg, 0.455 mmol: 405 μL of solution 100 mg/mL β-alanine and 86 μL/mL CF_3COOH in DMSO) and Et_3N (253 μL, 1.82 mmol) were added, and the mixture was stirred for 19 h at ambient temperature. The reaction mixture was evaporated in vacuum (oil pump) and dried. The residue was extracted in a mixture of $CHCl_3$ (4 mL), water (4 mL) and 2 M HCl (0.12 mL). Chloroform layer was washed with water (2 x 4 mL), evaporated, and the residue was dried in vacuum. The crude material was purified on silica gel column (~ 80 ml) in hexane/$CHCl_3$/2-propanol 15:5:1 by volume, elution with hexane/$CHCl_3$/2-propanol 15:5:1 + 0.5% AcOH by volume. After evaporation of fractions a residue was dissolved in chloroform, the solution was washed with water (2 x 3 mL), diluted with MeCN (3 mL) and evaporated. Thoroughly drying of the residue gave 105.5 mg (85%) of pure 2-azido-3-benzoyl-sphingosine-β-Ala, colorless syrupy glass.

TLC: R_f = 0.12 (hexane/$CHCl_3$/2-propanol 15:5:1 by volume), R_f = 0.49 ($CHCl_3$/2-propanol 4:1 by volume).

^{1}H NMR of 2-azido-3-benzoyl-sphingosine-β-Ala (700 MHz, [D]$CHCl_3$/[D_4]CH_3OH 1:1, 30°C): δ 8.210 (m, 2H; *orto*-H of Bz),7.766 (m, 1H; *para*-H of Bz),7.636 (m, 2H; *meta*-H of Bz), 6.114 (m, 1H; =CH), 5.754 (m, 2H; CH= and =C-CH-O), 4.383 (dd, J = 11.4, 4.6 Hz, 1H; OCH), 4.274 (dd, J

= 11.4, 7.7 Hz, 1H; OCH`), 4.172 (m, 1H; CH-N_3), 3.565 (t, J = 6.6 Hz, 2H; NCH_2 of βAla), 2.693 (t, J = 6.6 Hz, 2H; CH_2CO of βAla), 2.260 (q, J = 7.1, 7.1, 6.8 Hz, 2H; =C-CH_2), 1.565 (m, 2H; =C-C-CH_2), 1.440 (m, 20H; 10 CH_2), 1.043, (t, J = 7.1 Hz, 3H; CH_3) ppm.

2-Azido-3-benzoyl-sphingosine-β-Ala-ONSu

To a stirred solution of 2-azido-3-benzoyl-sphingosine-β-Ala (40 mg, 73.4 micromol) in 1,2-dichloroethane (1 mL) a solution of disuccinimidylcarbonate (37.6 mg, 147 micromol: 470 μL of 80 mg/mL solution in dimethyl formamide) and Et_3N (15.3 μL, 110 micromol) were added, and the mixture was stirred for 1.5 h at ambient temperature. The reaction mixture was acidified with AcOH (100 μL) and was placed on Sephadex LH-20 column (column volume 90 mL, eluent – $CHCl_3$/2-propanol 2:1 + 0.5% AcOH by volume). Fractions, containing pure 2-azido-3-benzoyl-sphingosine-β-Ala-ONSu were combined, evaporated, and the residue was dried in vacuum. Yield of 2-azido-3-benzoyl-sphingosine-β-Ala-ONSu was 42.4 mg (90%), white solid.

TLC: R_f = 0.56 (hexane/$CHCl_3$/2-propanol 15:5:1 by volume).

2-Azido-3-benzoyl-sphingosine-β-Ala-CMG(2)-biot

To a stirred suspension of biot-CMG(2) amine tri-Na salt (44 mg, 34.7 micromol) in dimethyl sulfoxide (2 mL) a solution of 2-azido-3-benzoyl-sphingosine-β-Ala-ONSu (24.5 mg, 38.2 micromol) in 1,2-dichloroethane (0.49 mL), water (0.7 mL) and 1 M aqueous $NaHCO_3$ (34.7 μL) were added, and the mixture was stirred for 2 h at ambient temperature. The reaction mixture was acidified with AcOH (6 μL) and placed on Sephadex LH-20 column (column volume 130 mL, eluent – water/MeOH/2-propanol/$CHCl_3$ 40:14:10:1 by volume). Fractions, containing pure 2-azido-3-benzoyl-sphingosine-β-Ala-CMG(2)-biot were combined, evaporated, the residue was dissolved in water (3 mL) and freeze-dried. Yield of 2-azido-3-benzoyl-sphingosine-β-Ala-CMG(2)-biot tetra-Na salt was 56,7 mg (90% on biot-CMG(2) amine), white solid.

TLC: R_f = 0.64 (CH_2Cl_2/EtOH/water 1:3:1 by volume), R_f = 0.40 (CH_2Cl_2/EtOH/water 1:3:1 + 2% AcOH by volume).

^{1}H NMR of 2-azido-3-benzoyl-sphingosine-β-Ala-CMG(2)-biot (700 MHz, $[D_2]H_2O/[D_4]CH_3OH$ 1:1, 30°C): δ 8.035 (m, 2H; *orto*-H of Bz), 7.653 (m, 1H; *para*-H of Bz), 7.507 (m, 2H; *meta*-H of Bz), 5.951 (m, 1H; =CH), 5.602 (m, 2H; CH= and =C-CH-O), 4.581 (dd, *J* = 7.9, 5.0 Hz, 1H; NHCH of biotin), 4.396 (dd, *J* = 7.9, 4.5 Hz, 1H; NHCH of biotin), 4.333-3.899 (total 35H; 4 CH_2COO, 12 NCH_2CO, CH_2O, CH-N_3), 3.469-3.351 (m, 6H; NCH_2CH_2N and CH_2N of βAla), 3.289 (m, 1H; NHCHCH of biotin), 2.985 (dd, *J* = 12.9, 4.8 Hz, 1H; NHCHCH of biotin), 2.761 (d, *J* = 12.9 Hz, 1H; NHCHCH of biotin), 2.554 (broad t, 2H; CH_2CO of βAla), 2.354 (m, 2H; $COCH_2$ of biotin), 2.086 (broad, 2H; =C-CH_2), 1.756 (m, 1H; $COCH_2CH_2CH_2CH$ of biotin), 1.677 (m, 2H; $COCH_2CH_2CH_2CH_2$ of biotin), 1.593 (m, 1H; $COCH_2CH_2CH_2CH$ of biotin), 1.462 (m, 2H; $COCH_2CH_2CH_2CH_2$ of biotin), 1.353 (m, 2H; =C-C-CH_2), 1.224 (m, 20H; 10 CH_2), 0.865 (t, *J* = 7.0 Hz, 3H; CH_3) ppm.
MS (MALDI-TOF): calc. for $C_{73}H_{108}N_{20}O_{27}S$ $Mw_{isotopic}$=1729, found 1704 [M($-N_2$+2H)+H], 1730 [M+H], 1752 [M+Na], 1768 [M+K].

2-N$_3$-Sphingosine-β-Ala-CMG(2)-biot

To a stirred solution 2-azido-3-benzoyl-sphingosine-β-Ala-CMG(2)-biot (49.1 mg, 27.01 micromol) in water (4.91 mL) methanol (9.82 mL) and Et_3N (0.737 mL) were added, and the mixture was kept for 77 h at ambient temperature. The reaction mixture was evaporated, the residue was thoroughly dried in vacuum and freeze-dried. The obtained 2-N_3-sphingosine-β-Ala-CMG(2)-biot was used subsequently without purification.
TLC: R_f = 0.34 (CH_2Cl_2/EtOH/water 1:3:1 + 2% AcOH by volume).
^{1}H NMR of 2-N_3-Sphingosine-β-Ala-CMG(2)-biot (700 MHz, $[D_2]H_2O/[D_4]CH_3OH$ 1:1, 30°C): δ 5.812 (m, 1H; CH=), 5.518 (m, 1H; =CH), 4.593 (dd, *J* = 7.9, 5.0 Hz, 1H; NHCH of biotin), 4.409 (dd, *J* = 7.9, 4.5 Hz, 1H; NHCH of biotin), 4.272-3.934 (total 35H; 4 CH_2COO, 12 NCH_2CO, CH_2O, CH-N_3), 3.720 (m, 1H; =C-CH-O), 3.459-3.352 (m, 6H; NCH_2CH_2N and CH_2N of βAla), 3.304 (m, 1H; NHCHCH of biotin), 2.998 (dd, *J* = 12.9, 4.9 Hz, 1H; NHCHCH of biotin), 2.769 (d, *J* = 12.9 Hz, 1H; NHCHCH of biotin), 2.557 (t, *J* = 6.5 Hz, 2H; CH_2CO of βAla), 2.360 (m, 2H; $COCH_2$ of biotin), 2.095 (m, 2H; =C-CH_2), 1.771 (m, 1H; $COCH_2CH_2CH_2CH$ of biotin), 1.696 (m, 2H;

$COCH_2C\underline{H}_2CH_2CH_2$ of biotin), 1.610 (m, 1H; $COCH_2CH_2CH_2C\underline{H}$ of biotin), 1.469 (m, 2H; $COCH_2CH_2C\underline{H}_2CH_2$ of biotin), 1.418 (m, 2H; =C-C-CH_2), 1.281 (m, 20H; 10 CH_2), 0.898 (t, J = 7.1 Hz, 3H; CH_3) ppm.

Sphingosine-β-Ala-CMG(2)-biot

To a stirred solution of 2-N_3-sphingosine-β-Ala-CMG(2)-biot (27.01 micromol from previous stage) in water (1.5 mL) methanol (4.5 mL), dithiothreitol (150 mg) and Et_3N (30 μL) were added, and the mixture was stirred for 48 h at ambient temperature. The reaction mixture was evaporated to dryness, the residue vas dissolved in 2 ml of water/2-propanol mixture (2:1 and placed on Sephadex LH-20 column (column volume 90 mL, eluent – water/2-propanol 2:1 by volume + 0.05 M Py•HOAc). Fractions, containing pure Sphingosine-β-Ala-CMG(2)-biot were combined, evaporated and the residue was dried in vacuum. Yield of sphingosine-β-Ala-CMG(2)-biot was 44.3 mg (89% on 2-azido-3-benzoyl-sphingosine-β-Ala-CMG(2)-biot, if calculated as tripyridunium salt), white solid.

TLC: 2-N_3-sphingosine-β-Ala-CMG(2)-biot R_f = 0.34; sphingosine-β-Ala-CMG(2)-biot R_f = 0.29 (CH_2Cl_2/EtOH/water 1:3:1 + 2% AcOH by volume), ninhydrin-positive.

Preparation of Ceramide-β-Ala-CMG(2)-biot

Scheme 1b.3

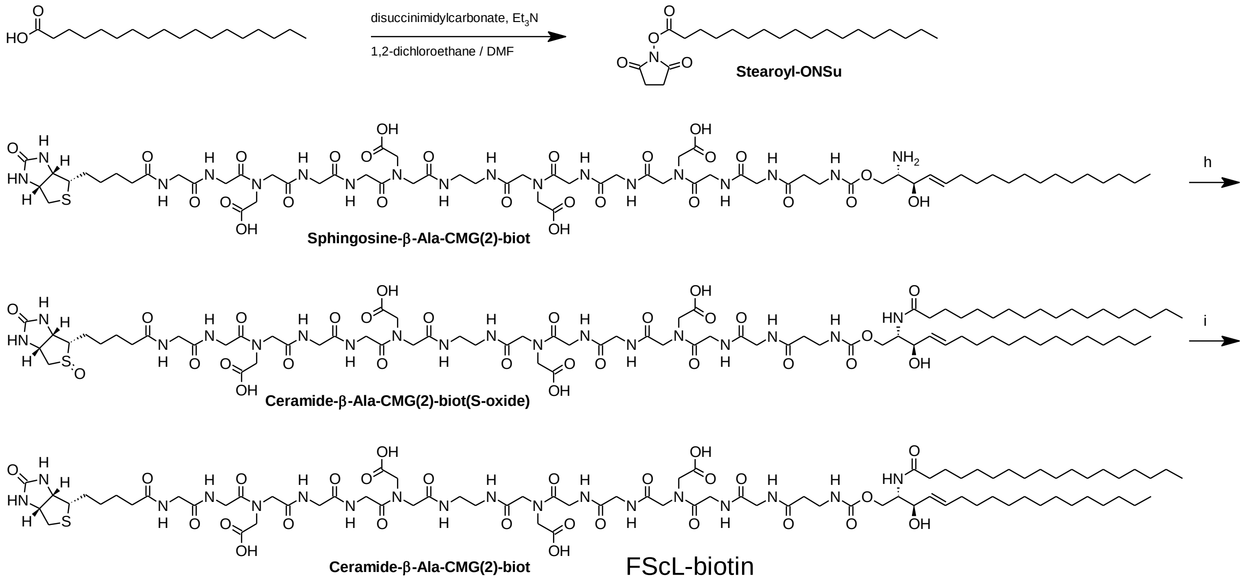

FScL-biotin

(h) Stearoyl-ONSu in 1,2-dichloroethane, aq. $NaHCO_3$, H_2O/2-propanol; (i) N-Methyl-mercaptoacetamide. H_2O, 69 h at 40 °C.

Stearoyl-ONSu

To a stirred solution of of stearic acid (76 mg, 0.27 micromol) in 1,2-dichloroethane (1 mL) and dimethyl formamide (1 mL) mixture disuccinimidylcarbonate (139 mg, 0.54 mmol) and Et_3N (37 μL, 0.27 mmol) were added. The reaction mixture was stirred for 40 min, acidified with AcOH (60 μL) and placed on Sephadex LH-20 column (column volume 130 mL, eluent – $CHCl_3$/2-propanol 2:1 by volume + 0.5% HOAc). Fractions, containing pure stearoyl-ONSu were combined, evaporated and the residue was dried in vacuum; yield 93 mg (91%), white solid.

TLC: stearic acid R_f = 0.49, stearoyl-ONSu R_f = 0.25 (hexane/EtOAc/AcOH 10:2:0.3 by volume).

Ceramide-β-Ala-CMG(2)-biot(S-oxide)

To a stirred solution of sphingosine-β-Ala-CMG(2)-biot (44.3 mg, 24.1 micromol) in a mixture of water (2 mL) and 2-propanol (3 mL) 1 M aqueous $NaHCO_3$ (160 μL) and a solution of N-oxysuccinimide ester of stearic acid (20.3 mg, 53 micromol) in 1,2-dichloroethane (0.27 mL) were added, and the

mixture was stirred for 8 h at ambient temperature. Additional portions of N-oxysuccinimide ester of stearic acid (20.3 mg, 53 micromol) in 1,2-dichloroethane (0.27 mL) and 1 M aqueous $NaHCO_3$ (160 μL) were added, and the mixture was stirred for 15 h at ambient temperature. The reaction mixture was acidified with AcOH (18 μL), evaporated, and the residue was dried in vacuum. The reaction products were separated on silica gel column (75 mL, prepared in $CHCl_3$/MeOH 4:1 by volume) eluted with $CHCl_3$/MeOH/water 2:6:1 by volume. Chromatography was accompanied and complicated with self-oxidation of biotin group into biotin(S-oxide). Repeated separation of ceramide-β-Ala-CMG(2)-biot, ceramide-β-Ala-CMG(2)-biot(S-oxide) and minor sphingosine-β-Ala-CMG(2)-biot(S-oxide) (oxidation of unreacted sphingosine-β-Ala-CMG(2)-biot) on silica gel column (75 mL, prepared in $CHCl_3$/MeOH 4:1 by volume, eluted with CH_2Cl_2/EtOH/water 1:2:1 + 1% Py) gave 20.5 mg (yield 45%) of pure ceramide-β-Ala-CMG(2)-biot(S-oxide).

TLC: ceramide-β-Ala-CMG(2)-biot R_f = 0.62, ceramide-β-Ala-CMG(2)-biot(S-oxide) R_f = 0.54, sphingosine-β-Ala-CMG(2)-biot(S-oxide) R_f = 0.46 (CH_2Cl_2/EtOH/water 1:3:1 + 1% Py by volume).

^{1}H NMR of ceramide-β-Ala-CMG(2)-biot(S-oxide) (700 MHz, $[D_2]H_2O/[D_4]CH_3OH$ 2:1, 30°C): δ 5.769 (m, 1H; CH=), 5.454 (m, 1H; =CH), under water (NHC<u>H</u> of biotin-S-oxide), 4.721 (dd, *J* = 8.9, 5.5 Hz, 1H; NHC<u>H</u> of biotin-S-oxide), 4.331-3.897 (total 36H; 4 CH_2COO, 12 NCH_2CO, CH_2O, CHN, =C-CH-O), 3.639 (d, *J* = 13.5, 1,9 Hz, 1H; NHCHC<u>H</u> of biotin-S-oxide), 3.446-3.344 (m, 7H; NCH_2CH_2N, CH_2N of βAla and NHCHC<u>H</u> of biotin-S-oxide), 3.219 (dd, *J* = 13.5, 6.7 Hz, 1H; NHCHC<u>H</u> of biotin-S-oxide), 2.536 (broad t, 2H; CH_2CO of βAla), 2.394 (t, *J* = 7.3 Hz, 2H; $COCH_2$ of biotin-S-oxide), 2.208 (broad t, 2H; $COCH_2$ of stearoyl), 2.030 (m, 2H; =C-CH_2), 1.901 (m, 2H; $COCH_2C\underline{H}_2$ of stearoyl), 1.734 and 1.593 (m, 6H; 3CH_2 of biotin-S-oxide), 1.304 (broad s, 50H; 25CH_2), 0.902 (t, *J* = 6.9 Hz, 3H; CH_3), 0.894 (broad t, *J* = 7.1 Hz, 3H; CH_3) ppm.

MS (MALDI-TOF): calc. for $C_{84}H_{140}N_{18}O_{28}S$ $Mw_{isotopic}$=1881, found 1882 [M+H], 1904 [M+Na], 1920 [M+K].

Ceramide-β-Ala-CMG(2)-biot (FScL-biotin)

To a stirred solution of ceramide-β-Ala-CMG(2)-biot(S-oxide) (20.5 mg, 10.89 micromol) in water (1.3 mL) N-methyl-mercaptoacetamide (373 μL) was added, and the mixture was kept for 69 h at 40 °C. The reaction mixture was placed on Sephadex LH-20 column (column volume 90 mL, eluent – water/2-propanol 2:1 by volume + 0.03 M HOAc + 0.06 M Py). Fractions, containing pure ceramide-β-Ala-CMG(2)-biot were combined, evaporated, and the residue was thoroughly dried in vacuum. The residue was dissolved in water (1 mL), titrated to pH 6.5 with 0.1 M $NaHCO_3$ and freeze-dried. Yield of ceramide-β-Ala-CMG(2)-biot tetra-Na salt was 15.8 mg (74%), white solid.

TLC: ceramide-β-Ala-CMG(2)-biot R_f = 0.64 (CH_2Cl_2/EtOH/water 1:3:1 by volume).

^{1}H NMR of ceramide-β-Ala-CMG(2)-biot (700 MHz, $[D_2]H_2O/[D_4]CH_3OH$ 2:1, 30°C): δ 5.764 (m, 1H; CH=), 5.443 (m, 1H; =CH), 4.603 (dd, *J* = 7.9, 4.9 Hz, 1H; NHC<u>H</u> of biotin), 4.423 (dd, *J* = 7.9, 4.6 Hz, 1H; NHC<u>H</u> of biotin), 4.312-3.964 (total 36H; 4 CH_2COO, 12 NCH_2CO, CH_2O, CHN and =C-CH-O), 3.444-3.352 (m, 6H; NCH_2CH_2N and CH_2N of βAla), 3.313 (m, 1H; NHCHC<u>H</u> of biotin), 2.997 (dd, *J* = 12.9, 4.8 Hz, 1H; NHCHC<u>H</u> of biotin), 2.778 (d, *J* = 12.9 Hz, 1H; NHCHC<u>H</u> of biotin), 2.552 (t, *J* = 6.5 Hz, 2H; CH_2CO of βAla), 2.365 (m, 2H; COCH_2 of biotin), 2.206 (broad t, 2H; COCH_2 of stearoyl), 2.039 (m, 2H; =C-CH_2), 1.759, 1.691, 1.601, 1.558 and 1.469 (m, total 8H; 6H of biot and COCH_2C<u>H</u>$_2$ of stearoyl), 1.390 (m, 2H; =C-C-CH_2), 1.299 (m, 48H; 24 CH_2), 0.905 (t, *J* = 6.6 Hz, 3H; CH_3), 0.895 (broad t, *J* = 7.0 Hz, 3H; CH_3) ppm.

MS (MALDI-TOF): calc. for $C_{84}H_{140}N_{18}O_{27}S$ $Mw_{isotopic}$=1865, found 1866 [M+H], 1888 [M+Na], 1904 [M+K].

Supplementary Note 1c. **Synthetic scheme and detailed protocol for synthesis of FSc^{1}L-biot (mimetic of FScL-biot)**

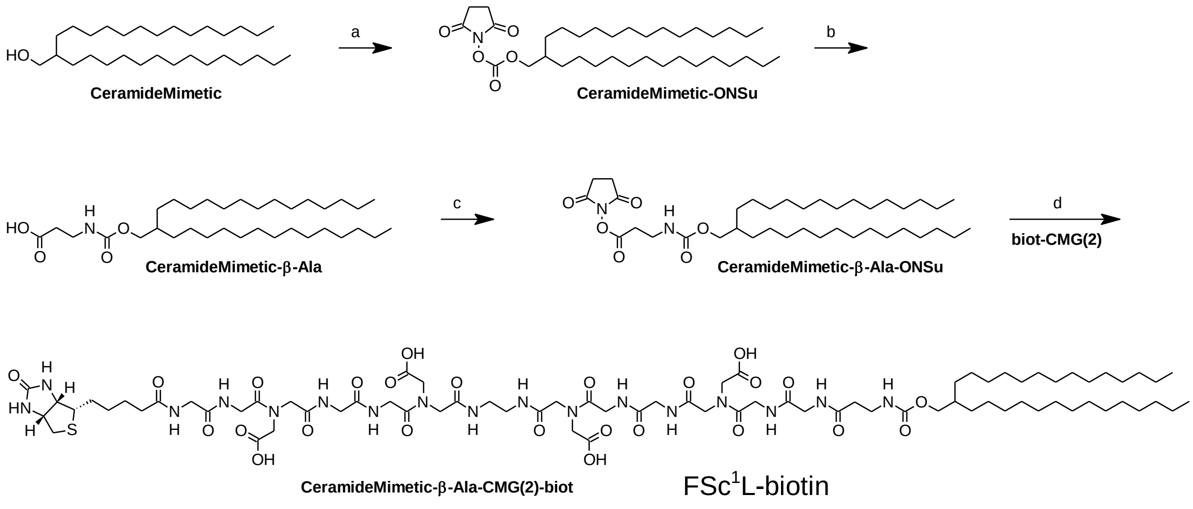

(a) disuccinimidylcarbonate, Et_3N, DMF/CH_2Cl_2 (b) β-alanine, Et_3N, DMSO/DMF/1,2-dichloroethane; (c) disuccinimidylcarbonate, Et_3N, DMF/1,2-dichloroethane; (d) biot-CMG(2) amine (see Supplementary Note 1b), aq. $NaHCO_3$, 1,2-dichloroethane/DMSO/H_2O.

CeramideMimetic-ONSu

To a stirred solution of CeramideMimetic (2-(tetradecyl)hexadecanol, *Katayama Chemical Industries Co., Ltd.,* 15.3 mg, 34.9 micromol) in a mixture of CH_2Cl_2 (1 mL) and dimethyl formamide (0.6 mL) a solution of disuccinimidylcarbonate (72 mg, 0.28 mmole) in dimethyl formamide (0.89 mL) and Et_3N (19.5 μL, 0.14 mmole) were added, and the mixture was stirred for 24 h at ambient temperature. The reaction mixture was acidified with AcOH (96 μL) and was placed on Sephadex LH-20 column (column volume 90 mL, eluent – $CHCl_3$/2-propanol 2:1 + 0.5% AcOH by volume). Fractions, containing pure CeramideMimetic-ONSu were combined, evaporated, and the residue was dried in vacuum. Yield of CeramideMimetic-ONSu was 19.1 mg (94%), white solid.

TLC: R_f = 0.35 (hexane/$CHCl_3$/2-propanol 15:5:1 by volume).

CeramideMimetic-β-Ala

To a stirred solution of CeramideMimetic-ONSu (19.1 mg, 32.9 micromol) in a mixture of 1,2-dichloroethane (1 mL) and dimethyl formamide (2 mL) a solution of β-alanine (10.6 mg, 119 micromol: 106 μL of solution 100 mg/mL β-alanine and 86 μL/mL CF_3COOH in DMSO) and Et_3N (66 μL, 0.47 mmol) were added, and the mixture was stirred for 17 h at ambient temperature. The reaction mixture was acidified with AcOH (60 μL), evaporated with 5 mL of 2-propanol to minimum volume and was placed on Sephadex LH-20 column (column volume 90 mL, eluent – $CHCl_3$/2-propanol 2:1 by volume + 0.05 M Py•AcOH). Fractions, containing pure CeramideMimetic-β-Ala were combined, evaporated, and the residue was dried in vacuum. Yield of CeramideMimetic-βAla was 17.4 mg (95%), white solid.

TLC: R_f = 0.38 (hexane/$CHCl_3$/2-propanol 2:4:1 by volume).

^{1}H NMR of CeramideMimetic-βAla (700 MHz, [D]$CHCl_3$/[D_4]CH_3OH 1:1, 30°C): δ 4.098 (d, *J* = 5.4 Hz, 2H; CH_2O), 3.549 (t, *J* = 6.5 Hz, 2H; CH_2N of βAla), 2.684 (t, *J* = 6.5 Hz, 2H; CH_2CO of βAla), 1.766 (m, 1H; $OCH_2C\underline{H}$), 1.433 (m, 52H; 26 CH_2), 1.046, (t, *J* = 7.1 Hz, 6H; 2 CH_3) ppm.

CeramideMimetic-β-Ala-ONSu

To a stirred solution of CeramideMimetic-β-Ala (11.3 mg, 20.4 micromol) in a mixture of 1,2-dichloroethane (1 mL) and dimethyl formamide (0.35 mL) a solution of disuccinimidylcarbonate (10.5 mg, 41 micromol: 131 μL of 80 mg/mL solution in dimethyl formamide) and Et_3N (4.3 μL, 31 micromol) were added, and the mixture was stirred for 2 h at ambient temperature. The reaction mixture was acidified with AcOH (100 μL) and was placed on Sephadex LH-20 column (column volume 90 mL, eluent – $CHCl_3$/2-propanol 2:1 + 0.5% AcOH by volume). Fractions, containing pure CeramideMimetic-β-Ala-ONSu were combined, evaporated, and the residue was dried in vacuum. Yield of CeramideMimetic-β-Ala-ONSu was 12.7 mg (96%), white solid.

TLC: R_f = 0.72 (hexane/$CHCl_3$/2-propanol 2:4:1 by volume).

CeramideMimetic-β-Ala-CMG(2)-biot (FSc¹L-biot)

To a stirred suspension of biot-CMG(2) amine tri-Na salt (22.3 mg, 17.6 micromol, described in Supplementary Note 1b) in dimethyl sulfoxide (1.5 mL) a solution of CeramideMimetic-β-Ala-ONSu (12.7 mg, 19.5 micromol) in 1,2-dichloroethane (0.25 mL), water (0.35 mL) and 1 M aqueous $NaHCO_3$ (35.2 μL) were added, and the mixture was stirred for 5 h at ambient temperature. The reaction mixture was acidified with AcOH (3 μL), evaporated with 3 mL of 2-propanol/water 1:1 mixture to minimum volume and was placed on Sephadex LH-20 column (column volume 90 mL, eluent – 2-propanol/water 1:2 + 3% CH_2Cl_2 + 0.3% Py by volume). Fractions, containing pure CeramideMimetic-β-Ala-CMG(2)-biot were combined, evaporated, and the residue was thoroughly dried in vacuum. The residue was dissolved in water (1 mL), titrated to pH 6.5 with 0.1 M $NaHCO_3$ and freeze-dried. Yield of CeramideMimetic-β-Ala-CMG(2)-biot tetra-Na salt was 29.2 mg (91% on biot-CMG(2) amine), white solid.

TLC: R_f = 0.61 ($CHCl_3$/CH_3OH/water 2:6:1 by volume).

^{1}H NMR of CeramideMimetic-β-Ala-CMG(2)-biot (700 MHz, $[D_2]H_2O/[D_4]CH_3OH$ 1:1, 30°C): δ 4.590 (dd, *J* = 7.9, 4.7 Hz, 1H; NHC$\underline{H}$ of biotin), 4.408 (dd, *J* = 7.9, 4.6 Hz, 1H; NHC$\underline{H}$ of biotin), 4.293-3.917 (total 34H; 4 CH_2COO, 12 NCH_2CO, CH_2O), 3.428-3.343 (m, 6H; NCH_2CH_2N and CH_2N of βAla), 3.302 (m, 1H; NHCHC$\underline{H}$ of biotin), 2.993 (dd, *J* = 12.9, 5.0 Hz, 1H; NHCHC$\underline{H}$ of biotin), 2.767 (d, *J* = 12.9 Hz, 1H; NHCHC$\underline{H}$ of biotin), 2.546 (t, *J* = 6.5 Hz, 2H; CH_2CO of βAla), 2.359 (m, 2H; $COCH_2$ of biotin), 1.768, (m, 1H; $COCH_2CH_2CH_2C\underline{H}$ of biotin), 1.695 (m, 2H; $COCH_2C\underline{H}_2CH_2CH_2$ of biotin), 1.609 (m, 2H; $COCH_2CH_2CH_2C\underline{H}$ of biotin and $OCH_2C\underline{H}$), 1.468 (m, 2H; $COCH_2CH_2C\underline{H}_2CH_2$ of biotin), 1.312 (m, 52H; 26 CH_2), 0.919, (t, *J* = 7.0 Hz, 6H; 2 CH_3) ppm.

MS (MALDI-TOF): calc. for $C_{78}H_{131}N_{17}O_{25}S$ Mw=1739, found 1740 [M+H], 1762 [M+Na], 1778 [M+K].

Supplementary Note 1d. **Synthetic scheme and detailed protocol for synthesis of FSsL-biot.**

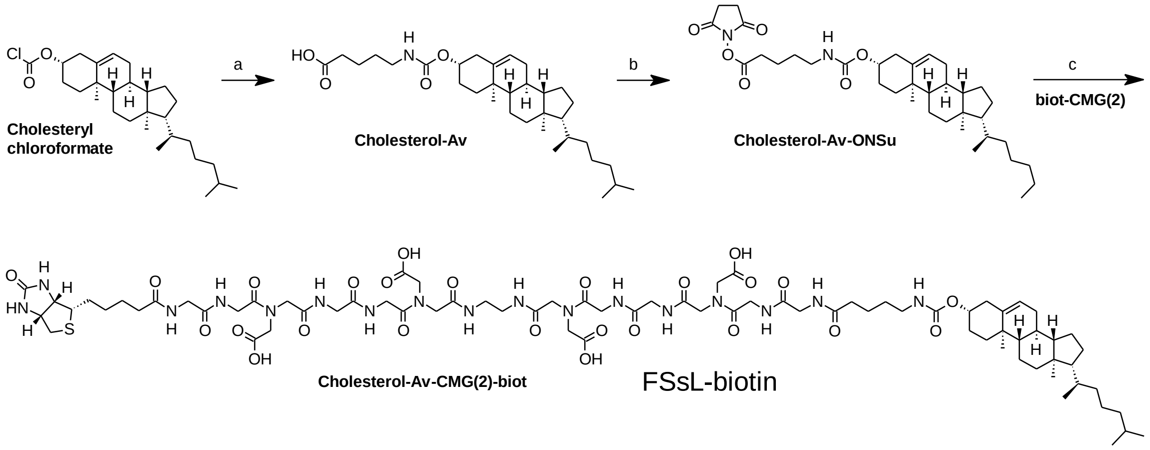

(a) 5-aminovaleric acid, $CHCl_3/H_2O$, $NaHCO_3$; (b) disuccinimidylcarbonate, Et_3N, DMF/1,2-dichloroethane; (c) biot-CMG(2) amine (see Supplementary Note 1b), aq. $NaHCO_3$, 1,2-dichloroethane/DMSO/H_2O.

Cholesterol-aminovaleric (AV) acid

To a rapidly stirred solution of 5-aminovaleric acid (117 mg, 1 mmol) and $NaHCO_3$ (252 mg, 3 mmol) in water (10 ml) a solution of cholesteryl chloroformate (449 mg, 1 mmol) in $CHCl_3$ (10 ml) was added. The mixture was stirred for 4 h at r.t., then acidified with 1 M HCl (2.5 ml) and chloroform layer was separated. After additional extraction of aqueous solution with chloroform (2 x 10 ml) combined organic extracts were washed with saturated NaCl and evaporated. The crude product was purified on silica gel column in $CHCl_3$/EtOAc 4:1 + 1% AcOH mixture as eluent. Yield of cholesteryl-carbonylaminopentanoic acid was 434 mg (82%), white solid.

TLC: $R_f = 0.35$ ($CHCl_3$/EtOAc 3:1 + 1% AcOH).

^{1}H NMR (700 MHz, [D]$CHCl_3$, 30°C): 9.95 (broad. s, 1H; COOH), 5.372 (m, 1H; =CH), 4.647 (m, 1H; NH), 4.490 (m, 1H; OCH), 3.187 (m, 2H; CH_2N), 2.387 (t, 2H; CH_2CO), 2.359 (m, 1H; =C-CH), 2.264 (m, 1H, =C-CH\`), 1.982 (m, 2H), 1.846 (m, 3H), 1.677 (m, 2H), 1.599-1.304 (m, 12H), 1.257 (m, 1H), 1.188-0.926 (m, 13H), 0.916 (d, *J*= 6.5 Hz, 3H; CH_3), 0.868 (d, *J*= 6.6 Hz, 3H; CH_3), 0.864 (d, *J*= 6.6 Hz, 3H; CH_3), 0.678 (s, 3H; CH_3) ppm.

Cholesterol-Av-ONSu

To a solution of cholesterol-AV (57 mg, 0.108 mmol) in the mixture of 1,2-dichloroethane (0.6 mL) and DMF (1.2 mL) disuccinimidyl carbonate (55 mg, 0.215 mmol) and Et_3N (15 μL, 0.108 mmol) were added. The solution was stirred for 30 min and then was acidified with AcOH (37 μL). Gel-permeation chromatography on Sephadex LH-20 (~ 90 mL of gel in $CHCl_3$/MeOH 2:1 + 0.5% AcOH), evaporation of fractions and drying of the residue in vacuum gave 67 mg (~ quantitative yield) of pure cholesteroylcarbonylaminopentanoic acid oxisuccinimide ester.

TLC: R_f = 0.29 (hexane/ethyl acetate/AcOH 10:4:1).

biot-CMG(2)-AV-cholesterol (FSsL-biot)

biot-CMG(2) amine tri-Na salt (68 mg, 0.0536 mmol, described in Supplementary Note 1b) was dissolved in water (3.8 mL) and diluted with 2-propanol (4.2 mL). To the intensively stirred solution a solution of cholesterol-AV-ONSu (67 mg, 0.107 mmol) in a mixture of 1,2-dichloroethane (1.1 mL) and DMF (3 mL) and then aqueous 1M $NaHCO_3$ (0.59 mL) were added. After 30 min of stirring (room temperature) 3 mL of mixture 2-propanol/water (1:1) was added, and the solution was stirred for additional 30 min. The reaction mixture was acidified with AcOH (68 μL), evaporated, and a residue was dried in vacuum. The obtained residue was washed with fresh-distillated Et_2O (3 x 8 mL, decantation) to remove excessive cholesterol-AV-ONSu and DMF. Gel-permeation chromatography of the crude product on Sephadex LH-20 (~ 170 mL of gel in 2-propanol/water 1:2), evaporation of fractions and drying of the residue in vacuum gave pure product. Freeze-drying of aqueous solution of product (2.5 mL of water) gave 86 mg (89% calc. on biot-CMG(2) amine) of biot-CMG(2)-AV-cholesterol tetra-Na salt.

TLC: R_f = 0.51 ($CHCl_3$/MeOH/water 2:6:1).

^{1}H NMR (700 MHz, $[D_2]H_2O/[D_4]CH_3OH$ 1:2, 30°C): 5.575 (m, 1H; =CH of cholesterol), 4.737 (dd, J = 7.8 Hz, J = 5 Hz, 1H; NHC$\underline{H}$ of biotin), 4.553 (dd, J = 7.8 Hz, J = 4.3 Hz, 1H; NHC$\underline{H}$ of biotin), 4.534 (m, 1H; OCH of cholesterol), 4.470-4.078 (total 32H; 4 CH_2COO, 12 NCH_2CO), 3.540 (m, 4H; NHC$\underline{H}_2$C$\underline{H}_2$NH), 3.446 (m, 1H; NHCHC$\underline{H}$ of biotin), 3.290 (t, J = 6.7 Hz, 2H;

CH_2N of aminovaleric res.), 3.149 (dd, *J* = 13 Hz, *J* = 5 Hz, 1H; $NHCHC\underline{H}$ of biotin), 2.916 (dd, *J* = 13 Hz, J‹2 Hz, 1H; $NHCHC\underline{H}$ of biotin), 2.515 (m, 6H; 2 CH_2CO and $=CH\text{-}C\underline{H}_2$ of cholesterol), 2.208-1.105 (total 39H; 29 H of cholesterol, 4H of aminovaleric res. and 6H of biotin), 1.105 (d, *J* = 6.6 Hz, 3H; $C\underline{H}_3CH$ of cholesterol), 1.050 and 1.046 (2 d, *J* = 6.6 Hz, 2x3H; 2 $C\underline{H}_3CH$ of cholesterol), 0.878 (s, 3H; CH_3 of cholesterol) ppm.

MS (MALDI-TOF): calc. for $C_{77}H_{119}N_{17}O_{25}S$ $Mw_{isotopic}$=1714, found 1737 [M+Na], 1759 [MNa+Na].

Supplementary references

1. Korchagina, E., Tuzikov, A., Formanovsky, A., Popova, I., Henry, S., Bovin, N. *Carbohydr. Res*. **356**, 238-246 (2012).

2. Gourier, Pincet, F., Perez, E., Zhang, Y., Zhu, Z., Mallet, J.-M., Sinaÿ, P. *Angewandte Chemie International Edition* **44,** 1683-1687 (2005).
