# Supplementary material for: Rapid one-step biotinylation of biological and non-biological surfaces
Source: Sci Rep. 2018 Feb 12;8:2845. doi: 10.1038/s41598-018-21186-3 (PMC5809608; doi:10.1038/s41598-018-21186-3)
Supplement: Supplementary file 2 — Supplementary Note 2 [file 41598_2018_21186_MOESM2_ESM.pdf]

Rapid one-step biotinylation of biological and non-biological surfaces

Stephen Henry[1*], Eleanor Williams[1], Katie Barr[1], Elena Korchagina[2], Alexandr Tuzikov[2], Natalia Ilyushina[3], Sidahmed A. Abayzeed[4], Kevin F. Webb[4], Nicolai Bovin[1,2*]

[1]AUT Centre for Kode Technology Innovation, School of Engineering, Computer & Mathematical Sciences, Auckland University of Technology, Auckland, New Zealand.

[2]Shemyakin & Ovchinnikov Institute of Bioorganic Chemistry, Russian Academy of Sciences, Moscow, Russian Federation

[3]FDA CDER, 10903 New Hampshire Avenue, Silver Spring, MD 20993, USA

[4]Optics & Photonics Research Group, School of Electrical & Electronic Engineering, University of Nottingham, United Kingdom

Supplementary Note 2. **Fluorescent- and visible staining of FSdL-biotin dots on coupon surfaces; staining of FSdL-biotin inkjet printed on wood surfaces**

Reagents:

FSdL-biotin, 0.2 mg

Coupon surfaces from Biosurface Technologies Corp (see **Table s2-1**)

2% BSA/PBS

1XPBS

Streptavidin-Alkaline Phosphatase, Sigma S2890, 1 mg/mL

NBT/BCIP substrate, Roche 11 681451 001

Substrate buffer, 50 mM Tris/150 mM NaCl/1 mM $MgCl_2 \cdot 6H_2O$

Streptavidin Alexa Fluor®488, Life Technologies S11223, 2 mg/mL.

Visible staining

Coupon preparation

Coupons used for staining were cleaned by soaking in Pyroneg solution (3 g/L) and sonicated for 10 min. Rinsed in water by sonication for 30 sec, then immersed in boiling methanol for 10 min. Rinsed again in water by sonication for 30 sec.

Individual coupons were hot-glued onto pipette tips for ease of handling.

Application of FSdL-biotin to surfaces for staining

FSdL-biotin was prepared at 2.4 mM with water by adding 40 μL water to 0.2 mg. Solutions of 5 μM and 50 μM were prepared by diluting the stock solution with water. Drops (1 μL) of FSdL-biotin at 5 μM and 50 μM were applied to the coupons at 12 and 6 o'clock positions respectively. Allowed to dry, then washed 3x with PBS.

Staining

Surfaces were flooded with 2% BSA/PBS to block against non-specific binding and left for 30 min at RT. Surfaces were flooded with Streptavidin Alkaline Phosphatase diluted 1:1000 in 2% BSA for 30 min at RT. Washed surfaces 6 times in 1x PBS and 1x Substrate buffer. Flooded surfaces with NBT/BCIP substrate diluted 1 in 50 in substrate buffer until sufficient precipitation occurred, ~10 min. Washed coupons in DI water and then dried.

Table s2-1 lists 33 surfaces, which gave positive staining results; example images of two materials are shown on **Fig. s2-1**a,b

Staining of wood surfaces inkjet printed with FSdL-biotin

Different varieties of wood were ordered from Carter Holt Harvey Wood Products NZ (195 Browns Rd, Manurewa, Auckland). Wood was inkjet printed with 50 μM FSdL-biotin using modified inkjet printer (Epson T21). Staining was carried out as above. Positive staining was observed on all wood surfaces tested, **Table s2-2**.

Fluorescent staining

Application of FSdL-biotin to surfaces for fluorescent staining

Drops (1 μL) of FSdL-biotin at 5 μM and 50 μM were applied to the coupons in 3 and 9 o'clock positions respectively. Allowed to dry, then washed 3x with PBS.

Fluorescent staining

Surfaces were flooded with 2% BSA/PBS to block against non-specific binding and left for 30 min at RT. Surfaces were flooded with Streptavidin Alexa Fluor® 488 diluted 1 in 200 in 2% BSA for 30 min at RT. Surfaces were washed 6 times 1x PBS. Observed under fluorescent microscope (Olympus BX51), x100 magnification, 1.903s exposure.

Table s2-1 lists 33 materials, which showed positive fluorescent staining; example images of two materials are shown in **Fig. s2-1c,d**.

Table s2-1| Positive visible and fluorescent staining of FSdL-biotin dots on coupons.

Part Number in*	Description
RD 128-PC	Polycarbonate
RD 128-316	Stainless Steel (316L)
RD 128-316P	Stainless Steel (316L) -Polished Surface
RD 128-HA	Hydroxyapatite
RD 128-GL	Borosilicate Glass
RD 128-PTFE	Teflon
RD 128-PL	Porcelain surface
RD 128-GT	Glazed Tile
RD 128-PS	Polystyrene
RD 128-PVC	PVC (white)
RD 128-PP	Polypropylene
RD 128-304	Stainless Steel (304)
RD 128-BUNA AHW	White High Grade FC Buna N Rubber
RD 128-PE	UMHW Polyethylene
RD 128-Ti	Titanium
RD 128-Ni	Nickel
RD 128-347	Stainless Steel (347)
RD 128-AL	Aluminium
RD 128-PG	Plexiglass (acrylic)
RD 128-PETG	Polyethylene Terephthalate Glycol (PETG)
RD 128-PU	Polyurethane (high temperature polymer)
RD 128-Vi	Viton
RD 128-CPVC	CPVC
RD 128-Si	Silicone Rubber Disc
RD 128-PEEK	Polyetherertherketone (PEEK:Arlon 1330)
RD 128-PET	Polyethylene Terephthalate (PET:Polyester, Dacron)
RD 128-Ny	Nylon Disc
RD 128-ABS	Acrylonitrile Butadiene Styrene (ABS)
RD 128-CC	Concrete Coated Polycarbonate (PC cup filled with concrete)
RD 128-NR	Natural Rubber Disc
RD 128-EPDM	EPDM Rubber Disc
RD 128-HY	Hypalon Rubber Disc
RD 128-PCC	Polycarbonate Cup

*source of coupons is http://biofilms.biz/biosurfacetechnologies.com/wp-content/uploads/2016/07/CBR-coupon-pricing-Jan2017.pdf

Table s2-2 | Positive visible staining of FSdL-biotin inkjet printed on wood surfaces

Wood veneers surfaces inkjet printed with FSdL-biotin	
Radiata Clear Pine	*(Pinus radiata)*
American Maple	*(Acer negundo)*
American Oak	*(Quercus macrocarpa)*
Totara	*(Podocarpus totara)*
American oak	*(Quercus macrocarpa)*
NZ Tawa	*(Beilschmiedia tawa)*
English Tawa	*(Aningeria spp)*
Tasmanian Ash	*(Eucalyptus regnans)*
Fijian Kauri	*(Agathis vitiensis)*
European Ash	*(Fraxinus excelsior)*

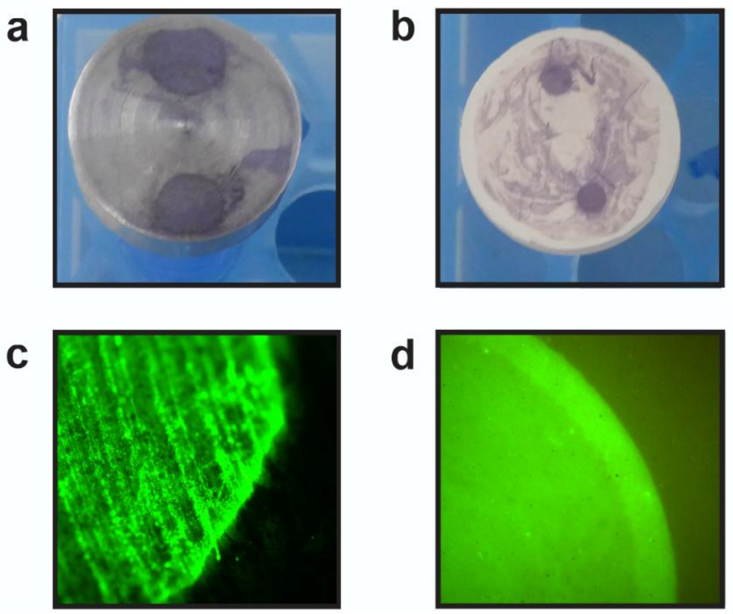

Figure s2-1 | Example images of FSdL-biotin dots on aluminium (**a, c**) and white high grade FC buna N rubber (**b, d**) coupons visualised by visible stain (**a, b**), or fluorescent staining (**c, d**).
