# Supplementary material for: Rapid one-step biotinylation of biological and non-biological surfaces
Source: Sci Rep. 2018 Feb 12;8:2845. doi: 10.1038/s41598-018-21186-3 (PMC5809608; doi:10.1038/s41598-018-21186-3)
Supplement: Supplementary file 3 — Supplementary Note 3 [file 41598_2018_21186_MOESM3_ESM.pdf]

Rapid one-step biotinylation of biological and non-biological surfaces

Stephen Henry[1*], Eleanor Williams[1], Katie Barr[1], Elena Korchagina[2], Alexandr Tuzikov[2], Natalia Ilyushina[3], Sidahmed A. Abayzeed[4], Kevin F. Webb[4], Nicolai Bovin[1,2*]
[1]AUT Centre for Kode Technology Innovation, School of Engineering, Computer & Mathematical Sciences, Auckland University of Technology, Auckland, New Zealand.
[2]Shemyakin & Ovchinnikov Institute of Bioorganic Chemistry, Russian Academy of Sciences, Moscow, Russian Federation
[3]FDA CDER, 10903 New Hampshire Avenue, Silver Spring, MD 20993, USA
[4]Optics & Photonics Research Group, School of Electrical & Electronic Engineering, University of Nottingham, United Kingdom

Supplementary Note 3. **Terminology guidelines for describing FSL constructs and resultant modified cells, viruses and surfaces.**

The term FSL is usually only used to describe function-spacer-lipid constructs that are dispersible in water and can spontaneously incorporate into cell membranes or coat surfaces. If the construct does not have these minimum features, it is not termed an FSL construct[13].

FSL constructs can bear a large variety of different functional heads[4,6-8,13] with each functional head capable of being carried by variations in both spacer design and lipid tails[4]. As a consequence, a single functional head can be presented as multiple FSL variations[4]. Furthermore, several FSL constructs can be added simultaneously (by creating a mixture of FSLs) to the same surface or membrane, and each with some degree of control over its relative concentration at the surface. In order to describe the FSL variations and the modifications they impart upon a surface (biological and non-biological) a standardized terminology has been adopted. These terms and their usage are consistent with recent publications.

FSL constructs

All constructs are generically referred termed as FSLs. The first aspect of the description is to provide a short name for the covalently attached functional (F) head group, for example FSL-biotin, or FSL-A_{tri} (e.g. blood group A trisaccharide), etc.

The second aspect is to describe the variations of spacer (S) used [4,5] . In this paper as only the CMG(2) spacer was used, the use of S for spacer was representative of all constructs.

The third aspect is to describe the lipid tail variations which were designated by a prefix to represent the lipid variation; with dL for DOPE (dioleoyl phosphatidylethanolamine); cL for ceramides; and sL for cholesterol. Where further variations exist within a lipid class exist, then a numeric suffix was used to signify these variations. With respect to structural variations of FSL-biotin constructs discussed in this paper (**Fig. 1**) the following terminology is used: FSdL, FScL, FSc^1L, and FSsL (details on the synthesis of these construct variations are shown in **Supplementary Notes 1**).

FSL Modified Surfaces

To distinguish FSL construct modified surfaces from other surface modifications the technique has been called Kode Technology[4,7,8,9,10,13]. The term "kode"derived from the technique name is then used to describe Kode Technology modified cells and surfaces, and FSL constructs are often also referred to as Kode constructs. The term kodecyte refers to any living (or dead) cell modified with FSL constructs[6,13], kodevirions to viruses modified with FSL constructs[7,8], and kodesomes to liposomes modified with FSL constructs. The term "koded"is the catch-all and generically refers to any biological or non-biological solution or surface modified with or containing FSL constructs.

Because multiple different biological and non-biological surface scan be modified with one or more of a large range of related and unrelated FSL variations, and with each at different concentrations, the terminology used includes multiple levels. As a general rule, the terminology used is the simplest form capable of differentiating the different koded surfaces or constructs used in a given setting (e.g. experiment or journal paper).

At the first level the solution containing the FSL construct(s)to be used to modify the surface is described. This simply relates to the short name and concentration of FSL(s) in the mix, preferably in µM. If a solution contains more than one FSL these are separated by a plus symbol, e.g. a solution containing FSL-biotin (50 µM) and FSL-A_{tri} (20 µM) could be described as FSL-biotin50+A_{tri}20.

At the second level the surface modified is described. For kodecytes the species and cell type used to make the kodecyte is described (but only if more than one cell variation is being used), followed by the FSL(s) used, and the FSL concentration in the solution used to prepare them. Therefore, if a kodecyte was made with murine red blood cells (mRBC) and FSL-biotin (50 µM) and FSL-A_{tri} (20 µM) then the resultant kodecytes could be described as an "mRBC biotin50+A_{tri}20-kodecytes". As kodecytes are always made with FSL constructs the term FSL is dropped from the terminology. It must be noted the concentration of FSLs in solution only creates standardised preparation of kodecytes if

the same conditions are used for the insertion process, and therefore the use of the solution concentrations to describe kodecytes is somewhat arbitrary.

Similarly to kodecytes, kodevirions, kodesomes and koded surfaces adopt a similar format based on describing the modified surface and the solution of FSL constructs used to achieve modification, e.g. biotin 50 koded stainless steel.

Secondary avidinylation.

Because FSL-biotin inserted into a membrane or coated on a surface can be stably modified by avidin, kodecytes prepared with FSL-biotin and subsequently secondarily modified with avidin can be called either avidin+biotin50-kodecytesor more simply just avidin-kodecytes if a standard FSL-biotin concentration is used. We use generic term avidin (and avidinylation) for all variations of avidin (e.g. egg avidin, streptavidin and neutravidin), unless more than one type of avidin is being used.
