# Supplementary material for: Rapid one-step biotinylation of biological and non-biological surfaces
Source: Sci Rep. 2018 Feb 12;8:2845. doi: 10.1038/s41598-018-21186-3 (PMC5809608; doi:10.1038/s41598-018-21186-3)
Supplement: Supplementary file 4 — Supplementary Note 4 [file 41598_2018_21186_MOESM4_ESM.pdf]

Rapid one-step biotinylation of biological and non-biological surfaces

Stephen Henry[1*], Eleanor Williams[1], Katie Barr[1], Elena Korchagina[2], Alexandr Tuzikov[2], Natalia Ilyushina[3], Sidahmed A. Abayzeed[4], Kevin F. Webb[4], Nicolai Bovin[1,2*]
[1]AUT Centre for Kode Technology Innovation, School of Engineering, Computer & Mathematical Sciences, Auckland University of Technology, Auckland, New Zealand.
[2]Shemyakin & Ovchinnikov Institute of Bioorganic Chemistry, Russian Academy of Sciences, Moscow, Russian Federation
[3]FDA CDER, 10903 New Hampshire Avenue, Silver Spring, MD 20993, USA
[4]Optics & Photonics Research Group, School of Electrical & Electronic Engineering, University of Nottingham, United Kingdom

Supplementary Note 4. **Generic precautions when processing FSL constructs**
Every surface that comes in contact with FSL constructs will rapidly (1 sec minimal contact) acquire an FSL-biotin coating; therefore, all consumables and equipment in contact with FSL solutions will become labelled and a small amount of material will be lost during experimental handling. Usually this loss is insignificant, but if handling requires exposure to significantly large surface areas, or very low concentrations of FSLs are used, then the losses may need to be accounted for. If high concentrations of FSL are applied to a solid surface layering of constructs will occur, and these upper layers may be lost during incubation and washing procedures (with released FSL's potentially able to label surrounding areas). There is no evidence for layering on biological lipid membranes. Optimization of FSL concentrations and methods (e.g. washing) for obtaining monolayers on surfaces is recommended.
Cleaning surfaces free of FSL constructs requires washing and the use of mixtures of solvents (<70% alcohol) and/or detergents[5]. Autoclaving (121°C for 20 min) does not usually destroy the FSL-biotin construct.
