# Supplementary material for: Rapid one-step biotinylation of biological and non-biological surfaces
Source: Sci Rep. 2018 Feb 12;8:2845. doi: 10.1038/s41598-018-21186-3 (PMC5809608; doi:10.1038/s41598-018-21186-3)
Supplement: Supplementary file 5 — Supplementary Note 5 [file 41598_2018_21186_MOESM5_ESM.pdf]

Rapid one-step biotinylation of biological and non-biological surfaces

Stephen Henry[1*], Eleanor Williams[1], Katie Barr[1], Elena Korchagina[2], Alexandr Tuzikov[2], Natalia Ilyushina[3], Sidahmed A. Abayzeed[4], Kevin F. Webb[4], Nicolai Bovin[1,2*]

[1]AUT Centre for Kode Technology Innovation, School of Engineering, Computer & Mathematical Sciences, Auckland University of Technology, Auckland, New Zealand.

[2]Shemyakin & Ovchinnikov Institute of Bioorganic Chemistry, Russian Academy of Sciences, Moscow, Russian Federation

[3]FDA CDER, 10903 New Hampshire Avenue, Silver Spring, MD 20993, USA

[4]Optics & Photonics Research Group, School of Electrical & Electronic Engineering, University of Nottingham, United Kingdom

Supplementary Note 5 **Coating of gold SPR chip with biot-CMG$_2$-DOPE, and interaction of modified chip with avidin**

To study the interaction of biot-CMG$_2$-DOPE with surfaces in real time, we used a bespoke surface plasmon resonance (SPR) instrument [1]. The instrument used in the experiment features a high sensitivity of ~1 x 10^{-7} refractive index units (RIU), providing a detection limit approximately two orders of magnitude superior to current commercial systems. The SPR sensing surface was a glass slide coated with a thin film of pure gold (50 nm). A custom flow cell/handling system was used to expose the bare gold to biot-CMG$_2$-DOPE (1:50 in PBS from 1mg/mL solution = 9.6 µM) at a flow rate of 500 µL/min (8.33 µL/s). The flow cell was perfused at this steady rate for 10 min, at which time the flow was halted and the SPR signal was monitored for 2 hours. Binding of biot-CMG$_2$-DOPE to the gold surface was measured at 10Hz, as an increase in refractive index at the gold surface - detectable as a shift in the SPR resonance angle (Figure s5-*1*). The rising phase of surface binding was fit, using MatLAB, to a monoexponential equation of the following form:

(Eq 1) $R = S(1 - e^{-\tau t})$ where *R* is the measured index (in RIU), *S* is the steady-state binding maximum, *t* is time, and *τ* is the binding rate constant.

For the binding of biot-CMG$_2$-DOPE to gold, the fitted time constant of binding at room temperature was 127.66s, reaching a steady state of 0.847x10^{-3} RIU by 360s. Binding remained extremely stable over more than 2 hours of continuous monitoring. The SPR sensor surface was then washed for 60 minutes with PBS, throughout which surface binding appeared stable.

Subsequently, to verify functional biotinylation, avidin ("Bio-Ultra", Sigma-Aldrich, 1.5x10^{-10} M in PBS) was added to the chamber at the same constant flow rate. Binding of avidin to the biot-CMG$_2$-DOPE coated surface was monitored for one hour, and the rising phase of the response (representing specific binding) was fit to Eq1 to extract a time constant of 230.78s, and a steady-state alteration in refractive index of 1.77x10^{-3}RIU.

[1] Abayzeed, S.A., et al. Responsivity of the differential-intensity surface plasmon resonance instrument. *Sensors and Actuators B: Chemical* **235**, 627-635 (2016):.

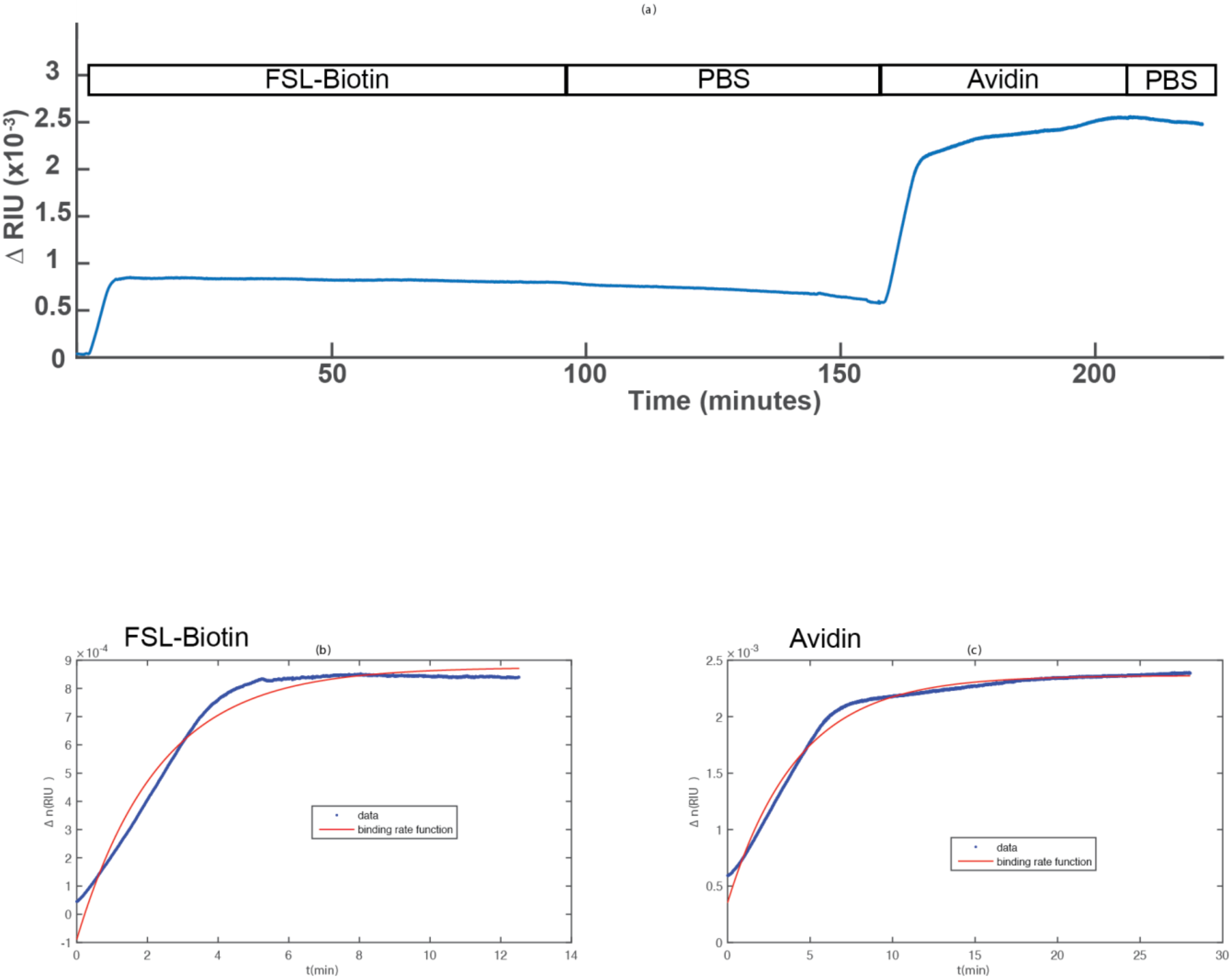

Figure s5-1. **Coating of gold SPR chip with biot-CMG$_2$-DOPE, and interaction of modified chip with avidin.** System configuration - from reference [1]: A differential-intensity surface plasmon resonance sensing platform was employed, adopting a prism-based Kretschmann-Raether configuration. A 633 nm linearly-polarised laser is coupled though a prism (BK7, n = 1.515) to the sensor-sample interface. A cylindrical lens is inserted into the beam path so that the light beam is focused into a line on the sensor chip. The angle of convergence of the light beam, inside the prism, is ∼±3°. Refractive index matching oil (Refractive Index 1.5150) was used between the prism and the sensor chip. The illumination resonance angle was set to ∼72° to excite surface plasmons on the surface of the chip. Since the incident angle is greater than the critical angle, total internal reflection occurs at the sensor–sample interface. The reflected light is collimated using a second cylindrical lens and detected using a CMOS camera (µEye, IDS GmbH, Germany). Signal processing was performed via MatLAB, using a differential-intensity analysis approach which is described in the cited reference.

Stock solutions: biot-CMG$_2$-DOPE (M_w=2079.28 g/mol, 1:50 in PBS from 1 mg/mL stock = 9.6 µM); avidin (M_w=66kDa, 1:100 in PBS from 1 mg/mL stock = 1.5×10^{-10} M).

(a) experimental curve for binding of biot-CMG$_2$-DOPE to the gold chip, followed by washing and binding of avidin to the formed biotinylated surface; (b) and (c) the respective fitted curves, which we contemplated placing together into an inset figure to show the comparison of rising phases.
